# Meiotic gatekeeper STRA8 suppresses autophagy by repressing *Nr1d1* expression during spermatogenesis in mice

**DOI:** 10.1371/journal.pgen.1008084

**Published:** 2019-05-06

**Authors:** Ianina C. Ferder, Leslie Fung, Yasuyo Ohguchi, Xiaoyu Zhang, Kara G. Lassen, Diane Capen, Dennis Brown, Ramnik J. Xavier, Ning Wang

**Affiliations:** 1 Vincent Center for Reproductive Biology, Massachusetts General Hospital, Harvard Medical School, Boston, MA, United States of America; 2 Department of Molecular and Integrative Physiology, University of Kansas Medical Center, Kansas City, KS, United States of America; 3 The Broad Institute of MIT and Harvard, Cambridge, MA, United States of America; 4 Center for Systems Biology, Massachusetts General Hospital, Harvard Medical School, Boston, MA, United States of America; 5 Center for Computational and Integrative Biology, Massachusetts General Hospital, Harvard Medical School, Boston, MA, United States of America; 6 Gastrointestinal Unit and Center for the Study of Inflammatory Bowel Disease, Massachusetts General Hospital, Harvard Medical School, Boston, MA, United States of America; Harvard Medical School, UNITED STATES

## Abstract

The transition from mitotic to meiotic cell cycles is essential for haploid gamete formation and fertility. *Stimulated by retinoic acid gene 8* (*Stra8*) is an essential gatekeeper of meiotic initiation in vertebrates; yet, the molecular role of STRA8 remains principally unknown. Here we demonstrate that STRA8 functions as a suppressor of autophagy during spermatogenesis in mice. *Stra8*-deficient germ cells fail to enter meiosis and present aberrant upregulation of autophagy-lysosome genes, commensurate with autophagy activation. Biochemical assays show that ectopic expression of STRA8 alone is sufficient to inhibit both autophagy induction and maturation. Studies also revealed that, *Nr1d1*, a nuclear hormone receptor gene, is upregulated in *Stra8*-deficient testes and that STRA8 binds to the *Nr1d1* promoter, indicating that *Nr1d1* is a direct target of STRA8 transcriptional repression. In addition, it was found that NR1D1 binds to the promoter of *Ulk1*, a gene essential for autophagy initiation, and that *Nr1d1* is required for the upregulated *Ulk1* expression in *Stra8*-deficient testes. Furthermore, both genetic deletion of *Nr1d1* and pharmacologic inhibition of NR1D1 by its synthetic antagonist SR8278 exhibit rescuing effects on the meiotic initiation defects observed in *Stra8*-deficient male germ cells. Together, the data suggest a novel link between STRA8-mediated autophagy suppression and meiotic initiation.

## Author summary

Meiotic initiation is a key feature of sexual reproduction that launches an intricate chromosomal program involving DNA double strand breaks (DSBs), homolog pairing, cohesion, synapsis, and recombination. Vertebrate gene *Stra8* is an essential gatekeeper of meiotic initiation. However, the molecular role of STRA8 and its target genes remain elusive. Using mouse spermatogenesis as a model, we report that STRA8 suppresses autophagy by repressing the transcription of a nuclear hormone receptor gene *Nr1d1*, and in turn, silencing the expression of *Ulk1*, a gene essential for autophagy initiation. Given that autophagy is critical for protein and cellular organelle recycling and for preventing genomic instability, our study suggests that this newly demonstrated function of STRA8, as a suppressor of autophagy, may be an important mechanistic feature of its role in meiotic initiation.

## Introduction

Meiosis is a fundamental process in sexual reproduction during which diploid cells halve their chromosome number by two rounds of cell divisions to generate haploid cells or gametes. In mammals, temporal regulation of meiosis in germ cells is sex-specific: meiosis in females begins during embryogenesis, whereas meiosis in males starts at puberty and persists throughout adulthood [1]. To enter meiosis, diploid cells must cease mitosis and then undergo one round of DNA replication, followed by the formation of DNA DSBs, meiotic chromosome pairing, cohesion, synapsis, and recombination. Although meiotic initiation has been extensively studied in some model organisms, such as yeast, flies, and worms, the mechanisms governing meiotic initiation in mammals remain elusive, largely because the molecular machinery controlling this process differs among species [2].

To date, the best characterized gatekeeper of meiotic initiation in vertebrates is *stimulated by retinoic acid gene 8* (*Stra8*) [3]. STRA8 is thought to act as a basic helix-loop-helix (bHLH) transcription factor, based on its DNA binding and transcriptional activity [4]. Interestingly, *Stra8* is expressed in a precise tissue-specific and developmental manner, whereby it is transitorily expressed only in premeiotic germ cells, of both sexes, shortly before their entry into meiosis [5, 6]. Functionally, *Stra8* likely governs both meiotic initiation and early meiotic progression. In one study, *Stra8*-deficient germ cells (exons 2–7 deleted) of both sexes do not display molecular hallmarks of meiotic initiation (e.g., DSBs) and fail to enter meiosis in juvenile mouse testes and in developing ovaries [7, 8]. Another study that used a different *Stra8*-deficient mouse line, in which exons 2–4 were deleted on an F1 hybrid (C57BL/6 x 129) background showed that *Stra8* functions instead in early meiotic prophase in spermatogenesis [9]. Nevertheless, *Stra8*-deficient germ cells of both sexes undergo meiotic arrest [7–9]. Thus, STRA8 is an essential regulator of meiosis that likely acts as a transcription factor, but currently there is little information on its molecular role and functional targets implicated in meiosis [10].

In this study, we report the unexpected finding that STRA8 acts as a suppressor of autophagy. Autophagy is a catabolic process involving self-digestion of protein and cellular organelles through lysosomes [11] and a major stress response pathway that promotes cellular survival by supplying nutrients and energy. In addition, autophagy plays a critical role in maintaining protein and cellular organelle quality control [12]. In recent decades, studies have identified genes that encode essential molecular factors of the autophagy pathway [11], including regulators of autophagy initiation (VPS34/PIK3C3, ULK1), nucleation (BECN1), elongation (ATG5-ATG12, ATG7, ATG16, and LC3B), and maturation through lysosome biogenesis (VPS18 and LAMP2). Moreover, TFEB has been identified as a master regulator of autophagy by inducing a broad spectrum of autophagy-lysosome genes [13]. Interestingly, lack of autophagy has been shown to instigate DNA damage events, including DNA DSBs, in somatic cells [14–16] (reviewed in ref. [17, 18]). The reported findings support a mechanism whereby STRA8 suppresses germ cell autophagy via direct transcriptional inhibition of a second transcription factor, NR1D1, which is needed for expression of the essential autophagy initiator ULK1. The data show that loss of *Nr1d1* expression or inhibition of NR1D1 function by its synthetic antagonist SR8278 exhibited rescuing effects on the meiotic initiation block observed in *Stra8*-deficient male germ cells. Together, our results suggest that STRA8-mediated autophagy suppression is a mechanistic feature of its role in meiotic initiation.

## Results

### *Stra8-*deficient testicular germ cells exhibit aberrant autophagy activation at the transcriptional level

**Fig 1:**
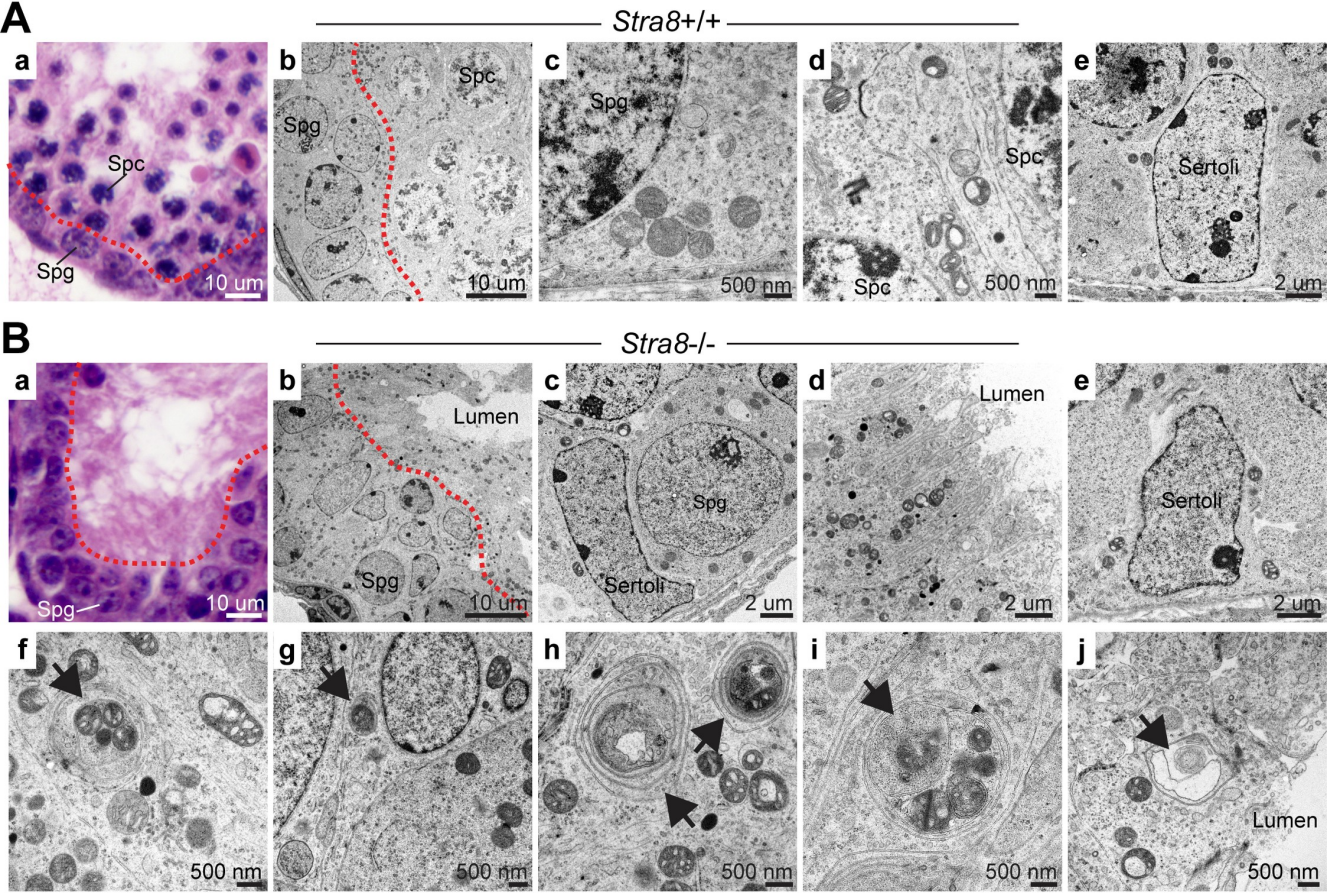
Transmission electron microscopy of wild-type and *Stra8*-deficient testes. (**A**, **B**) Testicular cross sections from wild-type (**A**) and *Stra8*-deficient (**B**) mice at 21 d.p.p.. (**Aa**, **Ba**) Hematoxylin-eosin-staining of testicular cross section from wild-type (**Aa**) and *Stra8*-deficient (**Ba**) mice. (**Ab**, **Bb**) Transmission electron microscopy of testicular cross section from wild-type (**Ab**) and *Stra8*-deficient (**Bb**) mice. Left of the red dashed line is the spermatogonia compartment (Spg). Right of the dashed line is the spermatocyte compartment (Spc). (**Ac-e**, **Bc-e**) Detailed views of spermatogonia (**Ac**, **Bc**), spermatocyte (**Ad**, **Bd**), and Sertoli cells (**Ae**, **Be**) in wild-type (**Ac-e**) and *Stra8*-deficient (**Bc-e**) testes. (**Bd**) A detailed view of the luminal compartment in *Stra8*-deficient testes. (**Bf-j**) Autophagosomes identified in *Stra8*-deficient testes (arrows).

To determine the molecular mechanism of STRA8-driven meiotic initiation, we used *Stra8*-deficient mice on a highly inbred C57BL/6 background [8]. We examined *Stra8*-deficient testes at 15–21 days postpartum (d.p.p.) by transmission electron microscopy. At this age, wild-type testes exhibited a normal presence of spermatogonia and meiotic spermatocytes (**Fig 1A, panels a and b**), whereas *Stra8*-deficient (*Stra8*^-/-^) testes exhibited a complete lack of meiotic spermatocytes despite the presence of spermatogonia (**Fig 1B, panels a-c**). Importantly, transmission electron microscopy revealed two novel characteristics in *Stra8*-deficient testes. First, the cellular integrity in the adluminal compartment of the seminiferous tubules appeared highly disrupted (**Fig 1B, panel b**), with the degenerative cells in this region having lost membrane integrity and erupted their cellular contents into the lumen (**Fig 1B, panel d**). Second, we frequently observed autophagosome structures (38 autophagosome structures observed in 200 germ cells from 2 *Stra8*-deficient testes), whereas in wild-type testes comparable autophagosome structures were not observed (0 autophagosome structures in 378 germ cells examined from 2 wild-type testes) (**Fig 1A, panel b and c**). Comparable autophagosome structures were not observed in Sertoli cells from both wild-type (**Fig 1A, panel e**) or *Stra8*-deficient testes (**Fig 1B, panel c and e**). This is consistent with a recent report that autophagy was not detected in spermatogonia, early spermatocytes and Sertoli cells in rat testes [19]. The autophagosome structures observed in *Stra8*-deficient germ cells were located in the cytoplasmic region of both non-degenerative (cells with intact membrane) (**Fig 1B, panels f-i**) and degenerative cells (**Fig 1B, panel j**). These double-membraned autophagosomes enclosed cellular organelles, including mitochondria (**Fig 1B, panels f—i**) and endoplasmic reticulum (**Fig 1B, panel j).** Together, our transmission electron microscopy study revealed aberrant autophagosome formation in *Stra8*-deficient germ cells.

**Fig 2:**
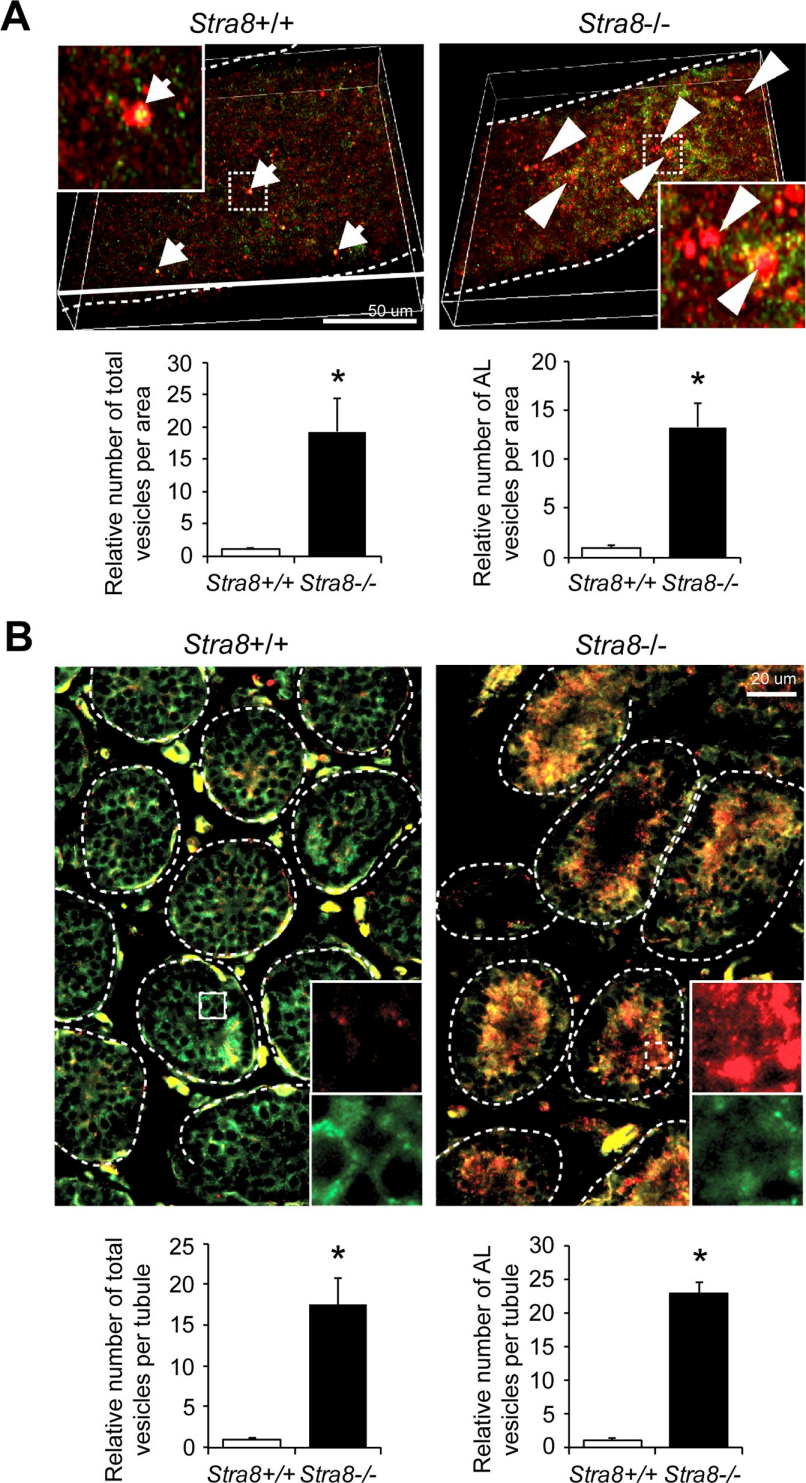
*In vivo* RFP-GFP-LC3 reporter in wild-type and *Stra8*-deficient testes. (**A**) Whole-mount seminiferous tubules from RFP-GFP-LC3 transgenic mouse testes in juvenile wild-type and *Stra8*-deficient backgrounds by confocal imaging (upper panels). Quantification of vesicle numbers per imaging area relative to wild-type samples is shown. Total, RFP-positive vesicles. AL (Autolysosome), RFP-positive GFP-negative vesicles. All values are means ± SD. n = 3 mice per genotype. *P < 0.05 (Student’s *t* test). (**B**) Testicular cross sections of RFP-GFP-LC3 transgenic mouse testes in juvenile wild-type and *Stra8*-deficient backgrounds. Dashed lines indicate seminiferous tubules. Arrows indicate autophagosome. Arrowheads indicates autolysosome (autophagosome maturation). Quantification of vesicle numbers per imaging area relative to wild-type samples is shown. Total, RFP-positive vesicles. AL (Autolysosomes), RFP-positive GFP-negative vesicles. All values are means ± SD. n = 3 mice per genotype. *P < 0.05 (Student’s *t* test).

During autophagy, autophagosomes serve as intermediate transport vesicles to target cellular components for degradation before their conversion into autolysosomes. Thus, aberrant appearance of autophagosomes in *Stra8*-deficient testes could result from either elevated autophagosome formation or impaired autophagosome turnover through fusion with lysosomes [20]. To distinguish between these possibilities, we examined autophagy activity in *Stra8*-deficient testes by using tandem fluorescent-tagged LC3 (RFP-GFP-LC3) by breeding RFP-GFP-LC3 transgenic allele into wild-type and *Stra8*-deficient backgrounds [21]. LC3 is a soluble protein and is distributed ubiquitously in cells. Upon autophagy activation, the cytosolic form of LC3 (LC3-I) is conjugated to phosphatidylethanolamine (PE) to form LC3-II, which is recruited to autophagosomal membranes, thereby serving as a well-characterized marker for autophagosomes [22]. In this reporter system, autophagosome vesicles (GFP-positive and RFP-positive puncta) can be distinguished from acidified autolysosome vesicles (GFP-negative and RFP-positive puncta) due to acidic quenching of the GFP signal, but not the RFP signal, after fusion with lysosomes. Whole-mount immunofluorescence imaging of seminiferous tubules from wild-type testes showed occasional merged GFP-positive and RFP-positive puncta, indicative of autophagosomes (**Fig 2A**). In contrast, seminiferous tubules from *Stra8*-deficient testes exhibited a significant increase in the number of both total vesicles (RFP-positive puncta) and autolysosome vesicles (GFP-negative and RFP-positive puncta) (**Fig 2A**). Direct fluorescence imaging of testicular cross sections confirmed a profound increase of autophagy in 98% of *Stra8*-deficient seminiferous tubules (**Fig 2B**). In contrast, 100% of the seminiferous tubules in wild-type testes exhibited diffuse GFP signal, suggesting cytoplasmic soluble LC3 and a lack of autophagosome formation (**Fig 2B, lower left panels**). To further confirm that the lack of autophagosomes in wild-type seminiferous tubules did not result from rapid turnover, wild-type and *Stra8*-deficient juvenile mice were treated with chloroquine, a weakly basic lysosomotropic agent that can block autophagosome fusion with lysosomes. Whereas chloroquine treatment resulted in an accumulation of autophagosome vesicles (GFP-positive and RFP-positive puncta) in *Stra8*-deficient testes, we did not observe an appreciable effect of autophagosome vesicle accumulation in wild-type testes, suggesting low autophagy activity (**S1 Fig**).

**Fig 3:**
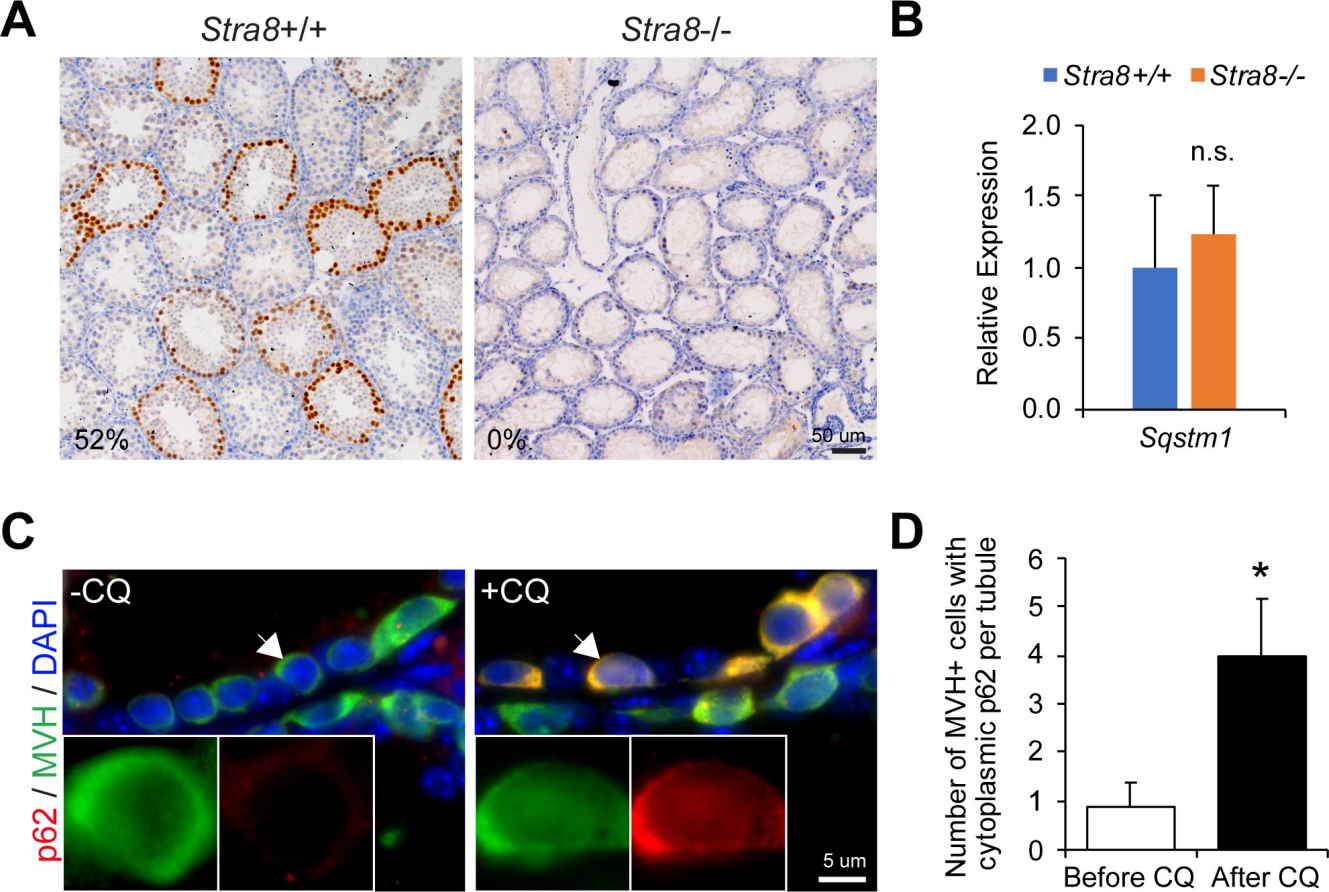
Rapid autophagic degradation of p62 in *Stra8*-deficient testicular germ cells. (**A**) Immunohistochemistry of p62 in wild-type and *Stra8*-deficient testes at 21 d.p.p.. Numbers indicate the percentages of seminiferous tubules containing germ cells with nuclear p62 accumulation. (**B**) qRT-PCR analysis of *Sqstm1* in wild-type and *Stra8*-deficient testes normalized to *β-actin*. Data represent mean ± SD; n = 3 per group. n.s.: not significant. (**C**) Dual immunofluorescence staining of p62 and MVH in *Stra8*-deficient testes treated with vehicle (PBS; left panel) or chloroquine (CQ; right panel) treatment for 7 days. (**D**) Quantification of the number of MVH+ germ cells exhibiting cytoplasmic p62 expression per seminiferous tubule with or without chloroquine treatment in *Stra8*-deficient testes. Data represent mean ± SD; n = 3 animals per group; 326 seminiferous tubules were examined from *Stra8*-deficient testes without chloroquine treatment. 296 seminiferous tubules were examined from *Stra8*-deficient testes with chloroquine treatment. *P < 0.05 (Student’s *t* test).

Autophagy is an essential intracellular degradation process. To evaluate autophagic degradation (flux) in wild-type and *Stra8*-deficient testes, we examined the protein level of p62 (or sequestosome 1, SQSTM1), a highly selective substrate for autophagic degradation [23]. The amount of p62 protein inversely correlate with autophagic flux activity: high levels of autophagy results in low p62 protein levels due to its degradation, while low levels of autophagy results in high p62 protein levels due to its accumulation. Wild-type testes contained seminiferous tubules with robust p62 protein accumulation at 21 d.p.p. (**Fig 3A**). In contrast, p62 protein level was almost completely lost in age-matched *Stra8*-deficient testes (**Fig 3A**). To determine if low p62 protein levels in *Stra8*-deficient testes were due to elevated autophagic degradation, possible changes due to *Sqstm1* gene (encoding p62) expression and autophagosome degradation (by chloroquine treatment) were evaluated. Quantification of *Sqstem1* mRNA showed comparable levels in age-matched wild-type and *Stra8*-deficient testes (**Fig 3B**) and whereas chloroquine showed no appreciable effect on p62 protein levels in wild-type testes (**S2 Fig**), chloroquine induced cytoplasmic p62 accumulation in germ cells of *Stra8*-deficient testes (**Fig 3C**). Together, these data suggest there is a rapid autophagic degradation of p62 in *Stra8*-deficient germ cells, reflective of high autophagic flux.

**Fig 4:**
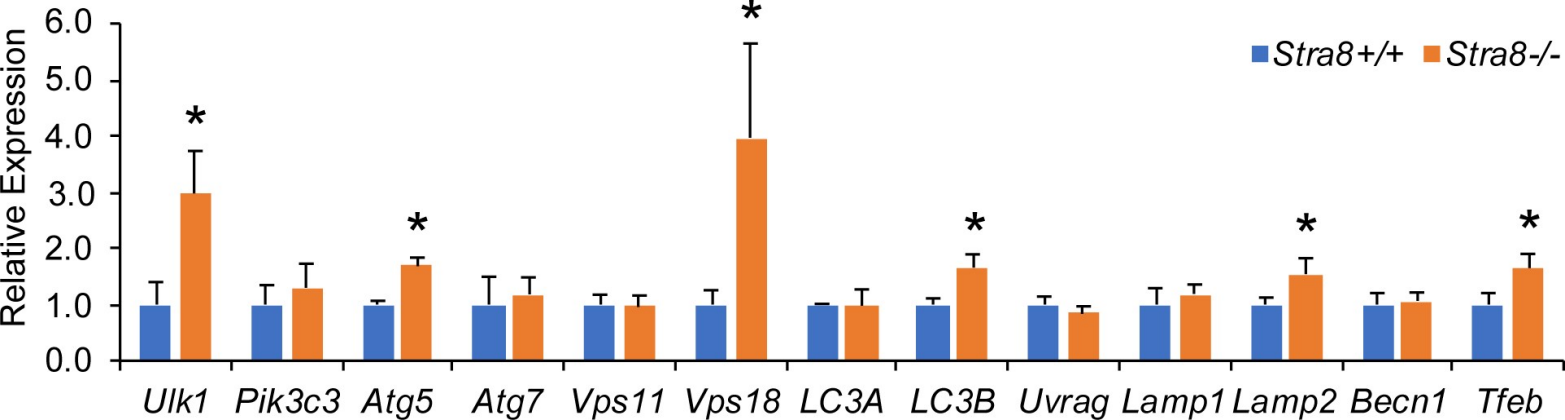
Transcriptional upregulation of selective autophagy-lysosome genes in *Stra8*-deficient testes. qRT-PCR analysis of autophagy and lysosome genes in wild-type and *Stra8*-deficient testes at 10 d.p.p. normalized to *β-actin*. Data represent mean ± SD; n = 5 per group. *P < 0.05 (Student’s *t* test).

To help uncover the mechanism by which STRA8 influences autophagy, expression levels of 14 essential autophagy-lysosome genes were evaluated by quantitative RT-PCR (qRT-PCR). For these studies, juvenile testes at 10 d.p.p. were used to assure that the germ cell content is comparable between wild-type and *Stra8*-deficient testes and, thus, observed differences between mRNA levels are not due to differences in germ cell numbers (**S3 Fig**). Among these 14 genes, 6 genes, namely, *Ulk1*, *Atg5*, *Map1lc3b*, *Vps18*, *Lamp2*, and *Tfeb*, were significantly upregulated in *Stra8*-deficient testes (**Fig 4**). These genes encode essential factors for autophagosome formation (ULK1, ATG5, Map1lc3b), lysosome function (VPS18, LAMP2), as well as a master regulator of autophagy-lysosome genes (TFEB). Together, these data suggest that induction of autophagy in *Stra8*-deficient testes results from upregulation of specific autophagy-lysosome gene expression.

### STRA8 inhibits autophagosome formation and maturation

**Fig 5:**
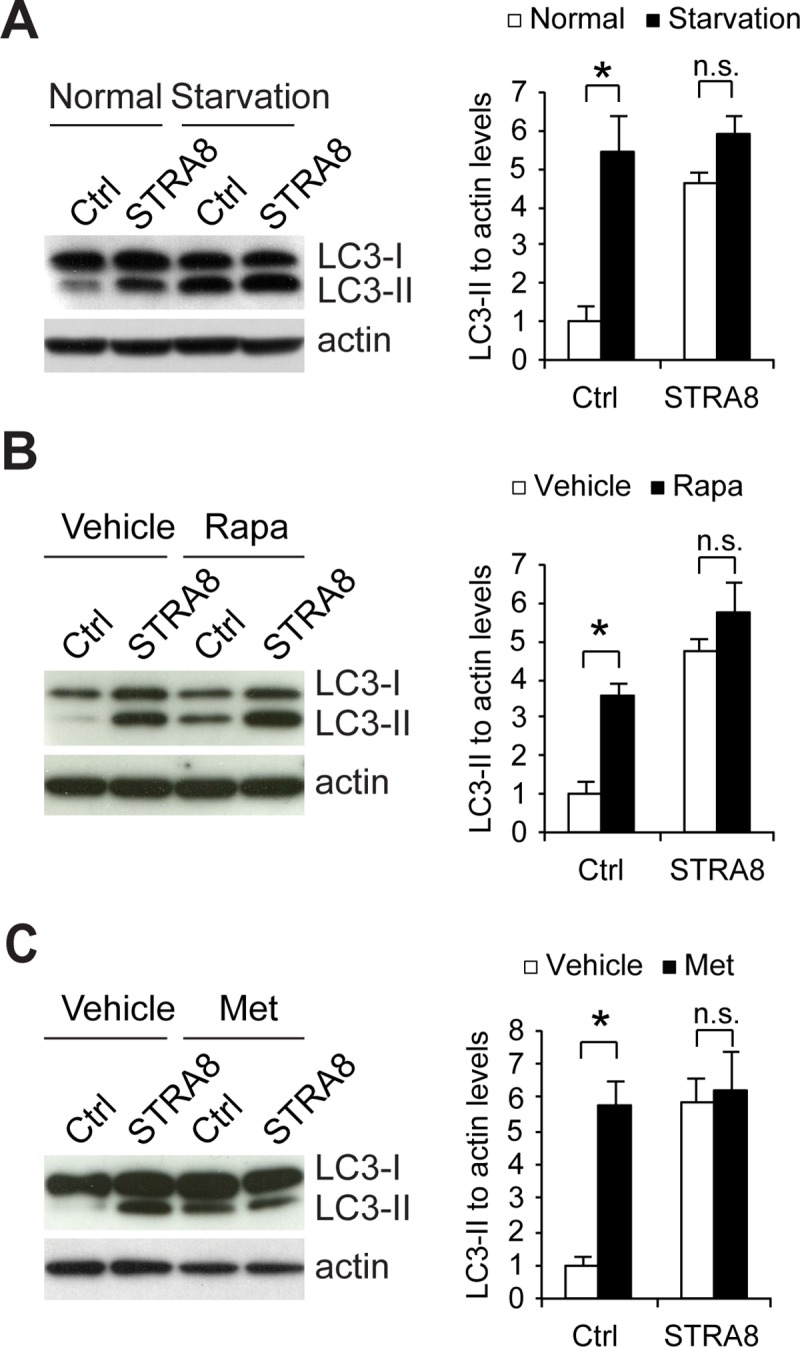
STRA8 inhibits *de novo* autophagosome formation upon autophagy induction. (**A**) Cell lysates from F9 cells stably expressing GFP (Ctrl) or STRA8 (tagged with GFP) treated with EBSS for 2 hours were subjected to Western blot analyses using antibodies as indicated. Graph shows quantification of LC3-II to actin ratio. Data represent mean ± s.e.m; n = 3 independent experiments; *P < 0.05 (Student’s *t* test). (**B**) Cell lysates from F9 cells stably expressing GFP (Ctrl) or STRA8 (tagged with GFP) treated with vehicle or rapamycin (Rapa; 0.1 μM) for 2 hours were subjected to Western blot analyses using antibodies as indicated. Graph shows quantification of LC3-II to actin ratio. Data represent mean ± s.e.m; n = 3 independent experiments; *P < 0.05 (Student’s *t* test). (**C**) Cell lysates from F9 cells stably expressing GFP (Ctrl) or STRA8 (tagged with GFP) treated with vehicle or metformin (Met; 2 mM) for 2 hours were subjected to Western blot analyses using antibodies as indicated. Graph shows quantification of LC3-II to actin ratio. Data represent mean ± s.e.m; n = 3 independent experiments; *P < 0.05 (Student’s *t* test).

Our data in *Stra8*-deficient testes suggests that STRA8 may suppress autophagy by inhibiting autophagy-lysosome gene expressions. Currently, there are no available STRA8-expressing germ cell lines and, because *Stra8* is transiently expressed on the verge of mitosis to meiosis transition, primary isolation and culture of *Stra8*-expressing cells could be challenging. Hence, to assess the role of STRA8 in autophagy suppression, STRA8 was ectopically and stably expressed in F9 embryonic carcinoma cells, a cell line regularly used for autophagy research [24]. Of note, autophagy machinery is present in every cell type and autophagy is an ongoing process in all cells; therefore, analysis of autophagy is often performed in cell lines [20]. To facilitate the identification of STRA8-expressing cells, STRA8 was tagged with GFP at its carboxyl terminus (**S4 Fig**). First, to test whether STRA8 suppresses autophagy induction, we used three commonly used inducers of autophagy, namely, amino acid starvation, rapamycin (mTOR inhibitor), and metformin (AMPK inducer) [25]. Autophagosome formation was detected by immunoblotting for LC3-II, which is a marker for autophagosomes [26]. It was found that, while LC3-II levels were significantly increased in control cells in all three conditions, there was no significant further increase of LC3-II levels in STRA8-expressing cells (**Fig 5**). These data suggest that STRA8 suppresses *de novo* autophagosome formation upon autophagy induction.

**Fig 6:**
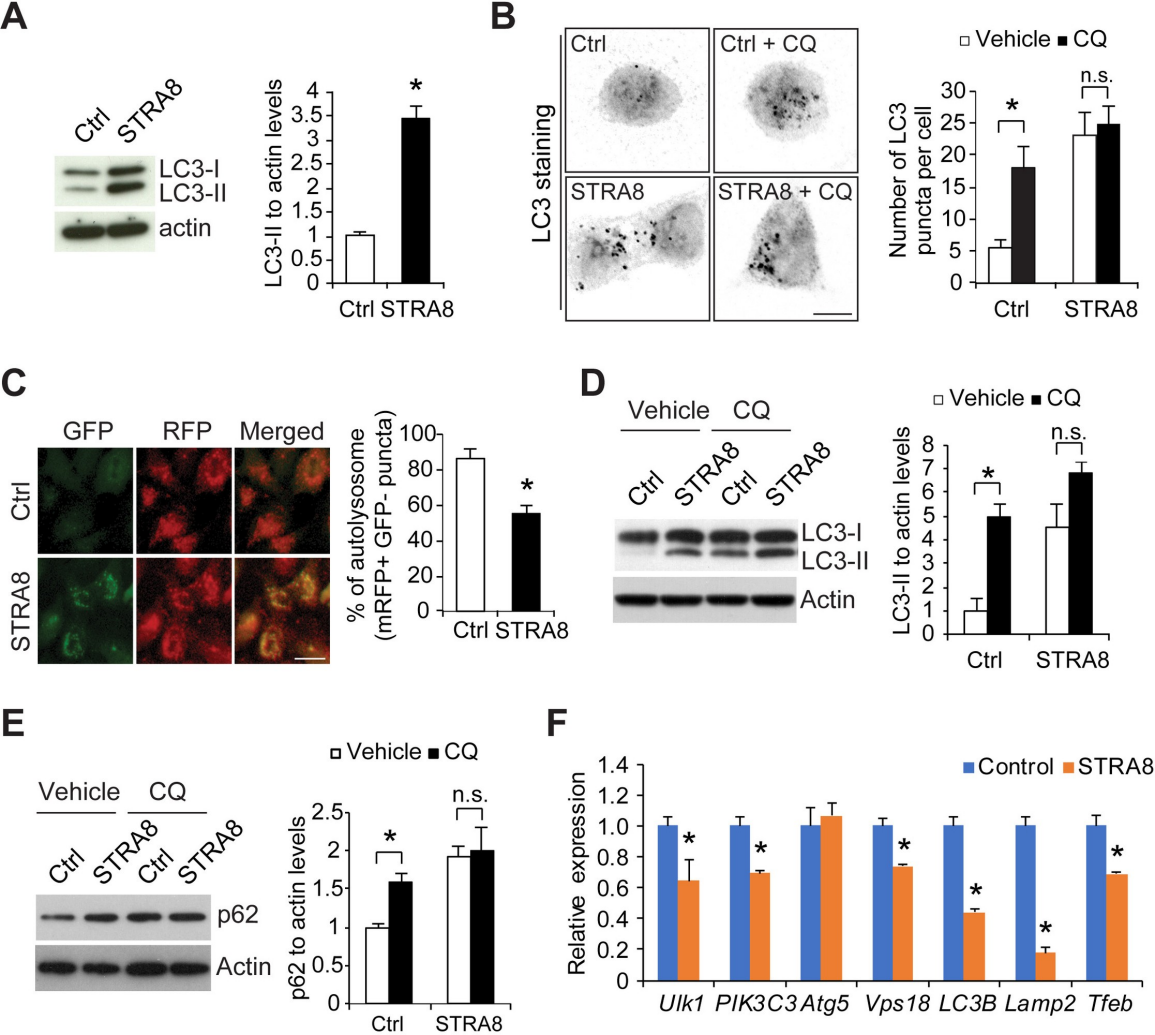
STRA8 inhibits autophagosome maturation under basal condition (no autophagy induction). (**A**) Cell lysates from F9 cells that stably express GFP (Ctrl) or STRA8 (tagged with GFP) at normal conditions were subjected to Western blot analyses by using antibodies as indicated. Graphs show quantification of LC3-II to actin ratio. Data represent mean ± s.e.m.; n = 3 independent experiments; *P < 0.05 (Student’s *t* test). (**B**) Fluorescence microscope images of F9 cells expressing GFP (Ctrl) and STRA8 stained with antibodies against LC3 treated with or without chloroquine for 2 hours. Graph shows quantification of LC3-positive puncta per cell. Black dots represent LC3 puncta. The number of LC3-positive puncta in each cell was counted for 100 cells in each group. Data represent mean ± s.e.m.; n = 3 independent experiments; *P < 0.05 (Student’s *t* test). (**C**) Representative fluorescence images of HeLa cells stably expressing GFP-mRFP-LC3 transfected with empty (Ctrl) or STRA8-expressing plasmid. Transfection efficiency of the plasmid into HeLa cells was approximately 80–85%. Graph shows the percentage of autolysosome vesicles (GFP-negative RFP-positive) in total vesicles (mRFP-positive). 100 cells were analyzed in each group. Data represent mean ± s.e.m.; n = 3 independent experiments; *P < 0.05 (Student’s *t* test). (**D**, **E**) Cell lysates from F9 cells that stably express GFP (Ctrl) and STRA8 (tagged with GFP) treated with vehicle or chloroquine (CQ; 20 μM) for 2 hours were subjected to Western blot analyses using LC3 (**D**) or p62 (**E**) antibodies. Graph shows quantification of LC3-II (**D**) or p62 (**E**) to actin ratio. Data represent mean ± s.e.m.; n = 3 independent experiments; *P < 0.05 (Student’s *t* test). (**F**) qRT-PCR analysis of autophagy and lysosome genes in control F9 cells and F9 cells expressing stably STRA8. Data represent mean ± SD; n = 3 cultures per group. *P < 0.05 (Student’s *t* test).

Although *de novo* autophagosome formation is impaired by STRA8 upon autophagy induction (**Fig 5**), we noted that there was a significant increase of LC3-II under basal condition (no autophagy induction) in STRA8-expressing cells, suggesting that STRA8 also inhibits autophagosome maturation, which results in autophagosome accumulation (upregulation of LC3-II) (**Fig 6A**). This result was confirmed at the cellular level by a significant increase of LC3 puncta (**Fig 6B**). Inhibition of autophagy flux frequently leads to autophagosome accumulation. Indeed, in our *in vitro* RFP-GFP-LC3 assay to monitor autophagy flux, STRA8 expression induced a significant accumulation of autophagosome vesicles (GFP-positive and RFP-positive puncta) that failed to mature into autolysosome vesicles (GFP-negative and RFP-positive puncta) (**Fig 6C**).

LC3-II and p62 are selectively degraded by autophagy. During chloroquine treatment, LC3-II and p62 accumulate due to inhibited autophagic flux, thereby serving as an indicator of autophagy flux activity. We show that the increment of p62 accumulation is significantly smaller in STRA8-expressing cells, suggesting that autophagy flux is being inhibited by STRA8. Consistent with the findings using *in vitro* RFP-GFP-LC3 assay, inhibition of autophagosome maturation by chloroquine resulted in a significant increase of LC3-II and p62 levels in control cells, but no significant further increase of LC3-II and p62 was observed in STRA8-expressing cells after chloroquine treatment (**Fig 6B, 6D and 6E**). Collectively, these data suggest that STRA8 also blocks autophagosome maturation under basal conditions.

To evaluate whether STRA8 influences autophagy-lysosome gene expression, the expression levels of the autophagy-lysosome genes that were upregulated in *Stra8*-deficient testes (**Fig 4**) were examined in these cells. We found that STRA8 expression alone was sufficient to cause a significant decrease in their expression levels, including *Ulk1*, *Pik3C3*, *LC3B*, *Vps18*, *Lamp2*, and *Tfeb* (**Fig 6F**). Together, these data suggest that STRA8 functions as a suppressor of autophagy by inhibiting autophagy-lysosome gene expression. Thus, loss of STRA8 function leads to the aberrant autophagy activation and upregulation of autophagy-lysosome gene expression in *Stra8*-deficient testes.

### Basic helix loop helix (bHLH) domain of STRA8 is required for autophagy suppression

STRA8 contains a highly conserved bHLH domain that exhibits DNA binding activity [4]. To gain mechanistic insight into STRA8-mediated autophagy suppression, two STRA8 mutants in the bHLH domain were generated (**S5A Fig**): in the first mutant, point mutations were introduced in the first helix domain, which are known to disrupt the DNA binding activity of bHLH family transcription factors (mHelix) [27]; in the second mutant, point mutations were introduced in the basic domain, which disrupts the nuclear localization of STRA8 (mNLS) [4]. Both STRA8 mutants exhibited impaired nuclear localization (**S5B Fig**) and lost their ability to suppress autophagy activation (**S5C Fig**) as well as maturation (**S5D Fig**). These data suggest that the bHLH domain of STRA8 is critical for its autophagy suppression function.

### *Nr1d1* is a genomic target of STRA8

**Fig 7:**
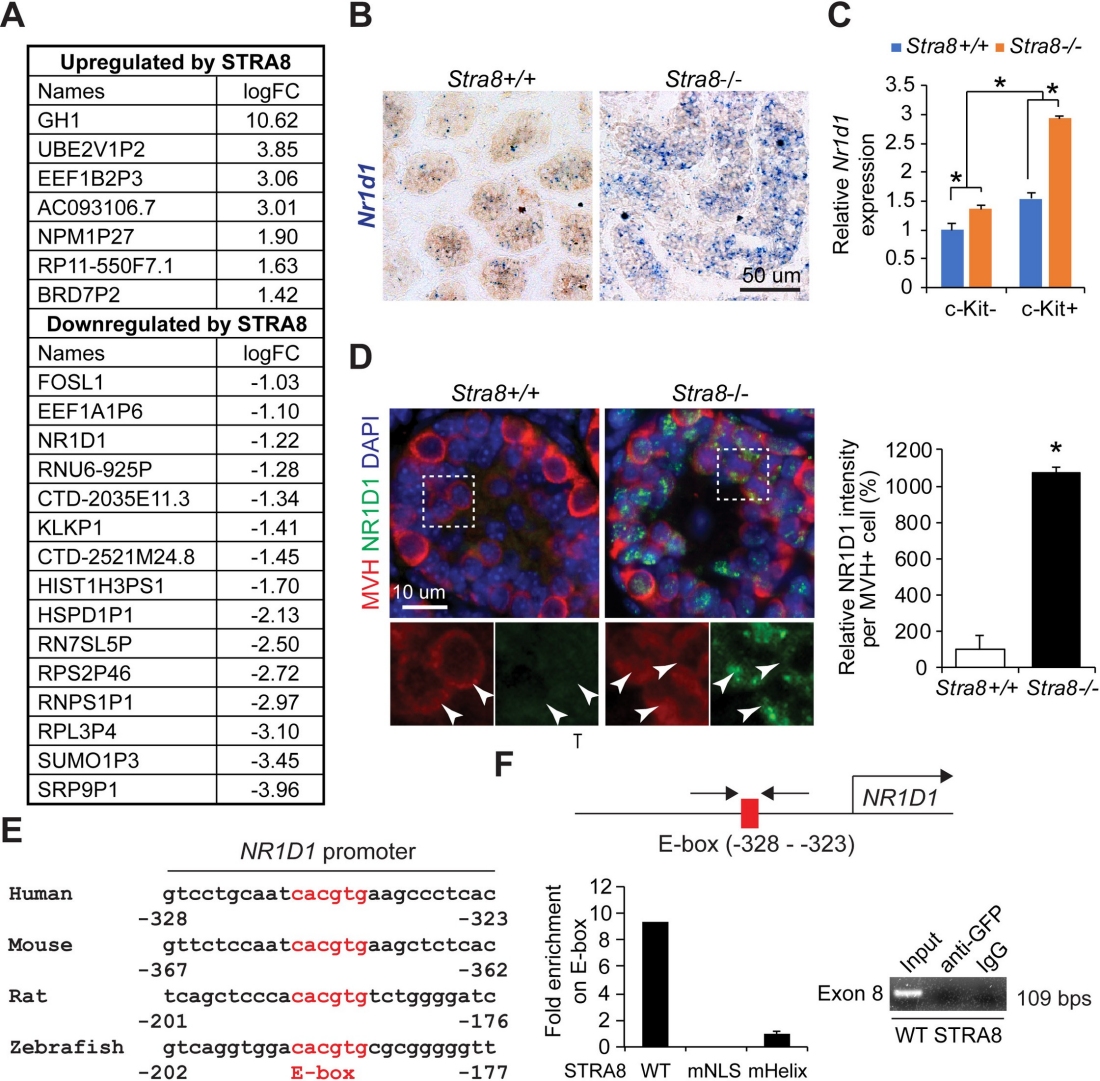
STRA8 represses *Nr1d1* expression. (**A**) Genes upregulated and downregulated by transient ectopic STRA8 expression detected by RNA-seq analysis. Total RNA was collected from cells transfected with empty vector (pCMV6) or STRA8 after 24 hours. (**B**) In situ hybridization of *Nr1d1* in testicular cross sections from age-matched wild-type and *Stra8*-deficient testes at 10 d.p.p.. (**C**) qRT-PCR analysis of *Nr1d1* expression relative to *β-actin* level in undifferentiated (c-Kit-negative integrin α6-high) and differentiating spermatogonia (c-Kit-positive integrin α6-low) isolated from juvenile wild-type and *Stra8*-deficient testes by FACS. Data represent mean ± SD; n = 3 mice per group; *P < 0.05 (Student’s *t* test). (**D**) Dual immunofluorescence staining of NR1D1 and MVH in wild-type and *Stra8*-deficient testes. Arrows in left panel indicate germ cells that do not express appreciable level of NR1D1 in wild-type testes. Arrows in right panel indicate germ cells in *Stra8*-deficient testes that express NR1D1. Note the germ cells in *Stra8*-deficient testes that express NR1D1 exhibit doublet nucleus, typical of what is frequently observed in preleptotene spermatocytes. A minimum of 60 cells in testicular cross sections of 2 mice was analyzed in each genotype. Data represent mean ± SD; *P < 0.05 (Student’s *t* test). (**E**) Schematic of the *NR1D1* promoter in human, mouse, rat, and zebrafish showing conserved E-box. (**F**) Upper, schematic of the primer set targeting the E-box in the human *NR1D1* promoter for ChIP analysis. Lower left, ChIP analysis of STRA8 WT, mNLS and mHelix binding to the *NR1D1* promoter at the E-Box in 293T cells after transient transfection. Graph represent mean ± SD from duplicate PCR reactions. Lower right, ChIP analysis shows absence of STRA8 WT binding to the exon 8 of *NR1D1* gene.

To identify putative target gene(s) of STRA8 that could mediate its autophagy suppression function, we have performed an RNA-sequencing analysis in cells with transient ectopic expression of STRA8 under normal conditions. STRA8 upregulated 7 genes and downregulated 15 genes (≥ 2-fold change) (**Fig 7A**). Interestingly, none of the autophagy and lysosome genes upregulated in *Stra8*-deficient testes was detected under this condition, suggesting that STRA8 regulates autophagy through other target(s). Among the regulated genes by STRA8, we found that *Nr1d1*, a gene downregulated by STRA8, encodes a nuclear hormone receptor also known as Rev-erb-α. *Nr1d1* is a critical circadian rhythm gene [28]. Recently, several studies have shown that NR1D1 acts as either an activator or an inhibitor of autophagy by regulating autophagy-lysosome gene expression, depending upon tissue context [29–31]. We therefore hypothesized that *Nr1d1* could be a functional target of STRA8.

We confirmed that ectopic expression of STRA8 significantly reduced *Nr1d1* levels in F9 cells and other cell types examined (**S6A and S6B Fig**), while the bHLH mutants of STRA8 failed to inhibit *Nr1d1* expression (**S6A Fig**). Reciprocally, *Stra8*-deficiency induced a significant upregulation of *Nr1d1* expression at mRNA level as detected by qRT-PCR analysis in testes (**S6C Fig**) and by in-situ hybridization (**Fig 7B**). To further evaluate if the induction of *Nr1d1* mRNA is intrinsic to germ cells, we isolated the c-Kit-positive integrin α6-low differentiating spermatogonia, in which *Stra8* is predominantly expressed, as well as the c-Kit-negative and integrin α6-high undifferentiated spermatogonia population, in which *Stra8* is yet to be fully activated (**S7 Fig**) [32]. We found that *Nr1d1* is more significantly upregulated in differentiating spermatogonia isolated from *Stra8*-deficient testes (**Fig 7C**). Moreover, we show that *Stra8*-deficient germ cells exhibited higher levels of NR1D1 expression at protein levels using immunofluorescence (**Fig 7D**).

To evaluate whether *Nr1d1* is a direct genomic target of STRA8, the proximal region of the *Nr1d1* promoter was examined for possible STRA8 binding sites. This identified a highly conserved canonical E-box (CAGCTG), the binding motif for members of the vertebrates bHLH protein family (**Fig 7E**). Chromatin immunoprecipitation (ChIP) detected robust STRA8 binding to this region of the *NR1D1* promoter (**Fig 7F**), suggesting that STRA8 represses *Nr1d1* transcription through E-box binding. Both bHLH mutants of STRA8 do not associate with the *NR1D1* promoter at this E-box. Together, these data suggest that *Nr1d1* is under direct transcriptional repression by STRA8.

### *Ulk1* is a genomic target of NR1D1 in mammalian testes

**Fig 8:**
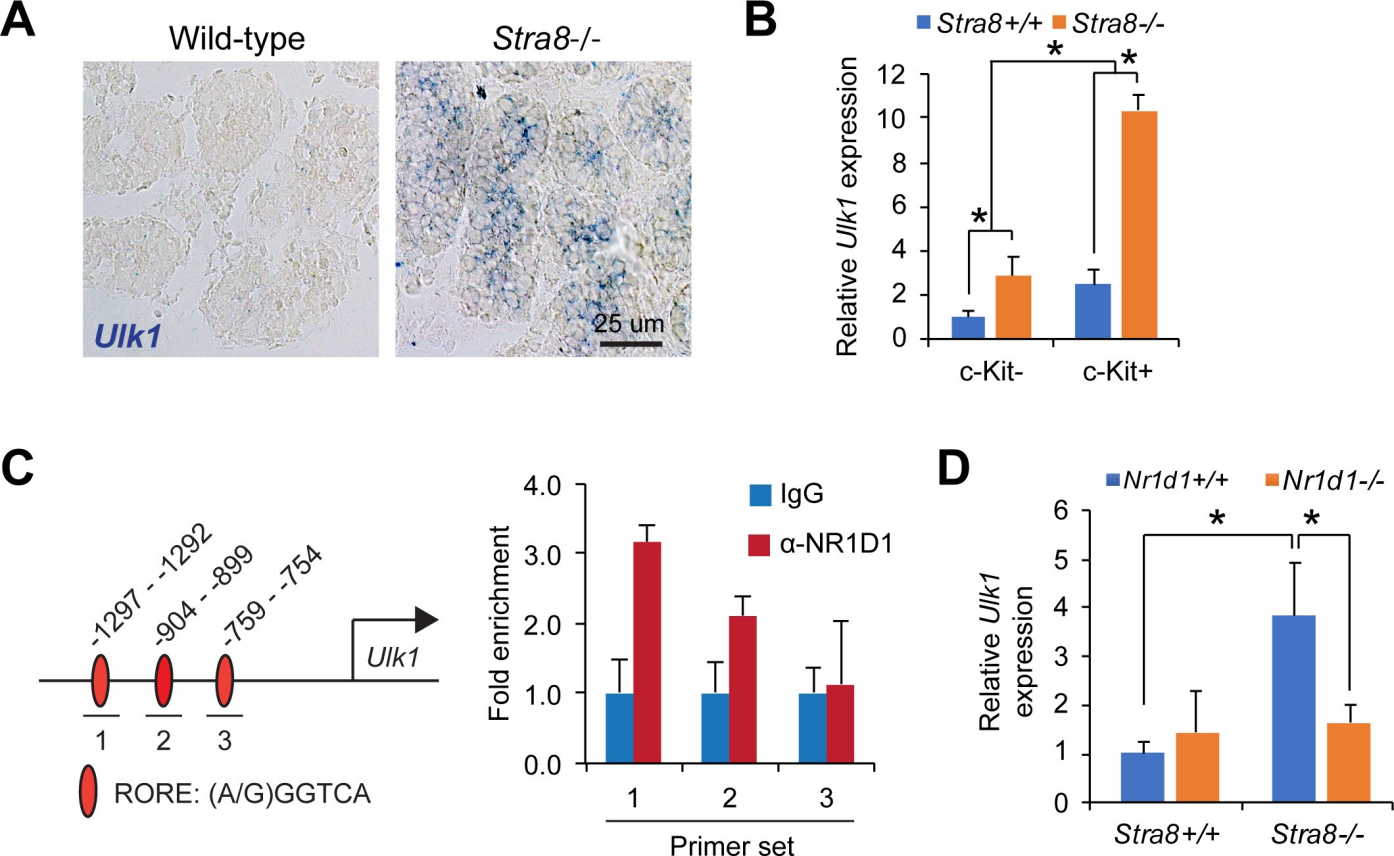
NR1D1 is required for the upregulated *Ulk1* expression in *Stra8*-deficient testes. (**A**) In situ hybridization of *Ulk1* in testicular cross sections from age-matched wild-type and *Stra8*-deficient testes at 10 d.p.p.. (**B**) qRT-PCR analysis of *Ulk1* expression relative to *β-actin* level in undifferentiated (c-Kit-negative integrin α6-high) and differentiating (c-Kit-positive integrin α6-low) spermatogonia isolated from juvenile wild-type and *Stra8*-deficient testes by FACS. Data represent mean ± SD; n = 3 mice per group; *P < 0.05 (Student’s *t* test). (**C**) Left panel, schematic of the primer sets targeting RORE sites in the mouse *Ulk1* promoter for ChIP analysis. Lower, ChIP analysis of NR1D1 binding to the *Ulk1* promoter at the RORE sites in mouse testicular lysates at 21 d.p.p.. Graph represent mean ± SD from triplicate PCR reactions. (**D**) qRT-PCR analysis of testicular *Ulk1* expression relative to *β-actin* levels in testes with indicated genotypes. Data represent mean ± SD; n = 3 mice per group; *P < 0.05 (Student’s *t* test).

To characterize whether STRA8 suppresses autophagy through NR1D1, we noted that past studies have shown that NR1D1 regulates autophagy through modulating the expression of *Ulk1* gene [29–31], which encodes an essential autophagy initiator [33, 34]. *Ulk1* mRNA is upregulated in *Stra8*-deficient testes, which we further confirmed using in-situ hybridization (**Fig 8A**). Moreover, we show that *Ulk1* expression was significantly increased in c-Kit-positive integrin α6-low differentiating spermatogonia isolated from *Stra8*-deficient testes (**Fig 8B**), concomitant with upregulation of NR1D1 expression (**Fig 7B–7D**). Moreover, we examined a 2-kb region upstream of the transcription start site of mouse *Ulk1* promoter and identified 3 RAR-related Orphan Receptor (ROR) DNA elements (ROREs), which consists of (A/G)GGTCA and could be putative NR1D1 binding sties [35, 36]. We therefore tested whether NR1D1 could directly regulate *Ulk1* expression in mouse testis by a ChIP assay. Importantly, we found that NR1D1 binding affinity declined progressively from the distal to the proximal ROREs of the *Ulk1* promoter (**Fig 8C**), suggesting that NR1D1 activates *Ulk1* expression by engaging directly on the distal ROREs.

Transcriptional activation of *Ulk1* is known to increase autophagy activity [37]. To test whether NR1D1 upregulation mediates the aberrant activation of *Ulk1* transcription in *Stra8*-deficient testes, *Ulk1* expression was evaluated in *Stra8*^-/-^;*Nr1d1*^-/-^ double knockout mice. Testicular *Ulk1* expression in double knockout mice was similar to that in wild-type, indicating that *Nr1d1* is required for *Ulk1* induction in *Stra8*-deficient testes (**Fig 8D**). Together, the results suggest that STRA8 suppresses autophagy by transcriptionally repressing *Nr1d1* expression and, consequently, inhibiting the expression of essential autophagy initiation gene *Ulk1*.

### Genetic and pharmacological NR1D1 inhibition is sufficient to rescue *Stra8*-deficient testicular germ cells from meiotic initiation arrest

**Fig 9:**
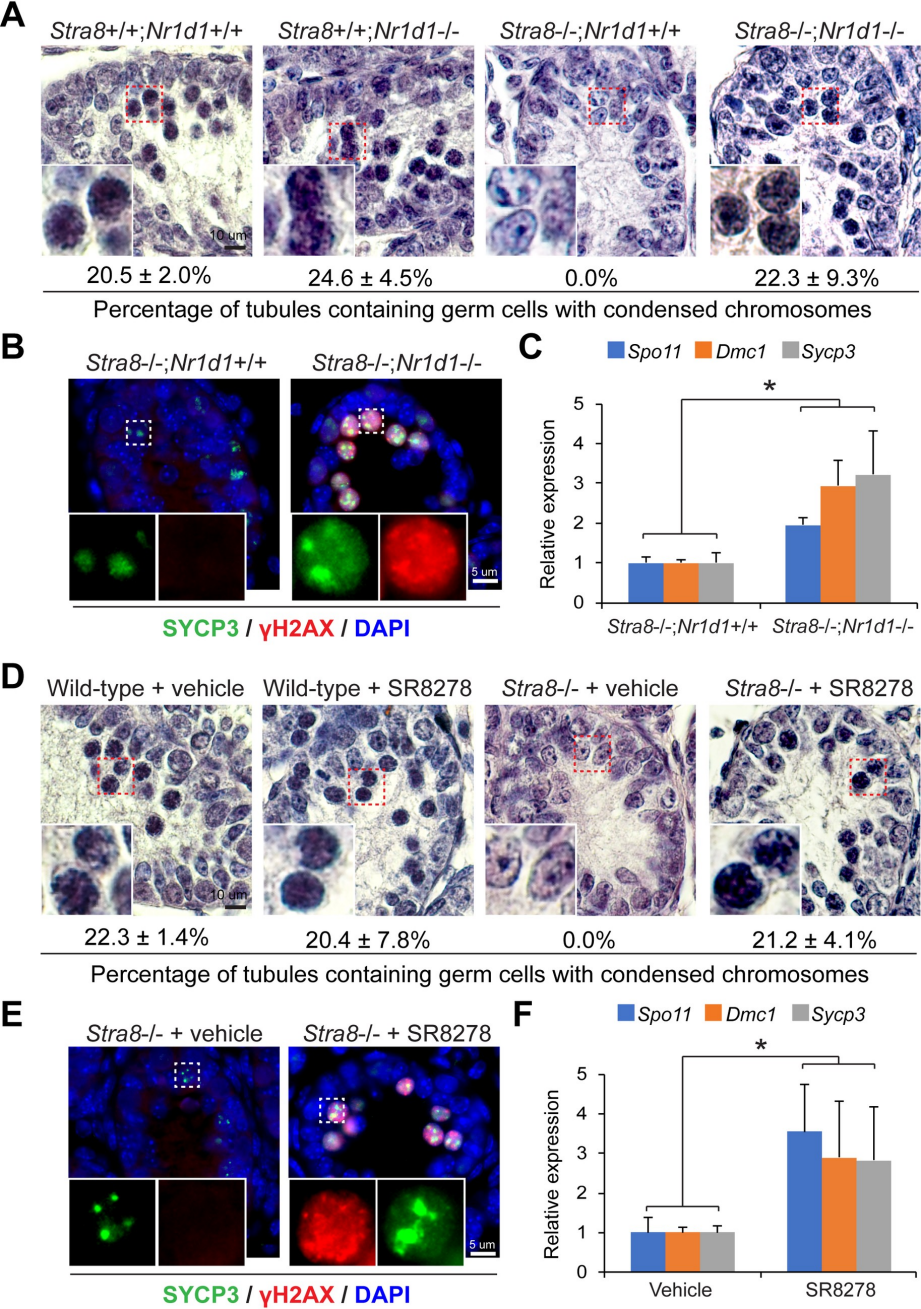
Genetic and pharmacological NR1D1 inhibition rescues meiosis initiation arrest in *Stra8*-deficient testes. (**A**) Photomicrographs of hematoxylin/eosin-stained testicular cross sections from testes with indicated genotypes at 10 d.p.p.. Insets show germ cells exhibiting chromosome condensation at early meiotic prophase. Percentages of tubules containing germ cells exhibiting chromosome condensation at early meiotic prophase are shown underneath. Data represent mean ± SD. n = 2–3 mice per genotype. (**B**) Dual immunofluorescence staining of γ-H2AX and SYCP3 in *Stra8*^-/-^;*Nr1d1*^+/+^ and *Stra8*^-/-^;*Nr1d1*^-/-^ testes at 10 d.p.p.. Note the brighter foci of SYCP3 staining in *Stra8*^-/-^;*Nr1d1*^+/+^ testes indicative of premeiotic status of their germ cells in contrast to the germ cells with nuclear distribution of SYCP3 and foci of γ-H2AX found in *Stra8*^-/-^;*Nr1d1*^-/-^ testes. (**C**) qRT-PCR analysis of testicular *Spo11*, *Dmc1*, and *Sycp3* expression normalized to *β-actin* from mice with indicated genotypes at 10 d.p.p.. Data are mean ± SD; n = 2–3 mice per group. *P < 0.05 (Student’s *t* test). (**D)** Photomicrographs of hematoxylin/eosin-stained testicular cross sections from wild-type and *Stra8*-deficient testes treated with vehicle or SR8278 (100 mg/kg) for 3 days. Testes were collected at 10 d.p.p.. Insets show germ cells exhibiting chromosome condensation at early meiotic prophase. Percentages of tubules containing germ cells exhibiting chromosome condensation at early meiotic prophase are shown underneath. Data represent mean ± SD. n = 3 mice per genotype. (**E**) Dual immunofluorescence staining of γ-H2AX and SYCP3 in *Stra8*-deficient testes treated with vehicle or SR8278. (**F**) qRT-PCR analysis of testicular *Spo11*, *Dmc1*, and *Sycp3* expression normalized to *β-actin* from mice treated with vehicle or SR8278. Data are mean ± SD; n = 4–5 mice per group. *P < 0.05 (Student’s *t* test).

To test whether repression of *Nr1d1* activation by STRA8 is required to initiate meiosis, we evaluated whether NR1D1 inhibition could rescue *Stra8*-deficient testicular germ cells from meiotic initiation arrest. *Stra8*-deficient germ cells fail to enter meiosis during the first round of meiotic initiation in juvenile mouse testes [8]. Thus, we evaluated meiotic initiation of testicular germ cells into leptotene spermatocytes at 10 d.p.p.. In agreement with the previous report [8], we found that while spermatocytes at early meiotic prophase (leptotene) have appeared as a result of the first round of meiotic initiation in wild-type testes, these meiotic spermatocytes were absent in *Stra8*-deficient testes at this age (**Fig 9A and 9D**). Consistently, germ cells exhibiting nuclear distribution of SYCP3 (a synaptomeal complex protein) [38] together with foci of γ-H2AX (a hallmark of DNA DSBs) [39], two molecular characteristics of leptotene spermatocytes, were absent in *Stra8*-deficient testes (**Fig 9B and 9E**). Furthermore, gene expression analysis showed that testicular levels of *Spo11*, which encodes a topoisomerase essential for meiotic DSB formation [40, 41], *Dmc1*, which encodes a recombinase functioning in meiotic DSB repair [42, 43], and *Sycp3*, were significantly downregulated in *Stra8*-deficient mice (**S8 Fig**). Therefore, these missing hallmarks of meiotic initiation in *Stra8*-deficient testes at 10 d.p.p. provide a platform to evaluate the potential rescuing effects of NR1D1 inhibition on meiotic initiation.

Notably, juvenile testes from *Stra8*^-/-^;*Nr1d1*^-/-^ mice at 10 d.p.p. contained germ cells with nuclear morphology resembling that of leptotene spermatocytes as observed in wild-type testes (**Fig 9A; S9A Fig**). Consistently, immunostaining revealed germ cells that exhibit nuclear distribution of SYCP3 together with foci of γ-H2AX staining in these testes (**Fig 9B**). Testicular levels of *Spo11*, *Dmc1*, and *Sycp3* were significantly upregulated in *Stra8*^-/-^;*Nr1d1*^-/-^ mice when compared to *Stra8*^-/-^;*Nr1d1*^+/+^ mice (**Fig 9C**). Moreover, *Nr1d1* knockout alone showed no appreciable effect on meiotic initiation (**Fig 9A; S9B and S9C Fig**). Taken together, these results suggest that genetic loss of *Nr1d1* exhibited rescuing effects on the meiotic initiation arrest in *Stra8*-deficient testicular germ cells.

To further examine the effect of NR1D1 inhibition on meiotic initiation arrest in *Stra8*-deficient testicular germ cells, we treated *Stra8*-deficient mice with SR8278, a synthetic NR1D1 antagonist [44]. Consistent with the results of genetic NR1D1 inhibition, we found that pharmacological inhibition recovered the appearance of germ cells with nuclear morphology as well as molecular hallmarks of leptotene spermatocytes in *Stra8*-deficient testes (**Fig 9D and 9E**). In addition, SR8278 treatment significantly stimulated testicular levels of *Spo11*, *Dmc1*, and *Sycp3* in *Stra8*-deficient testes (**Fig 9F**). SR8278 treatment showed no appreciable effects on meiosis in wild-type testes (**Fig 9D; S9D and S9E Fig**). Taken together, the results from both genetic and pharmacological inhibition of NR1D1 suggest that aberrant upregulation of NR1D1 contributes to the meiotic initiation arrest of *Stra8*-deficient germ cells and that inhibition of *Nr1d1* expression is an important feature of STRA8-directed meiotic initiation.

### Aberrant autophagy activation and upregulation of autophagy-lysosome gene expression in *Stra8*-deficient fetal ovaries during the developmental window of meiotic initiation

**Fig 10:**
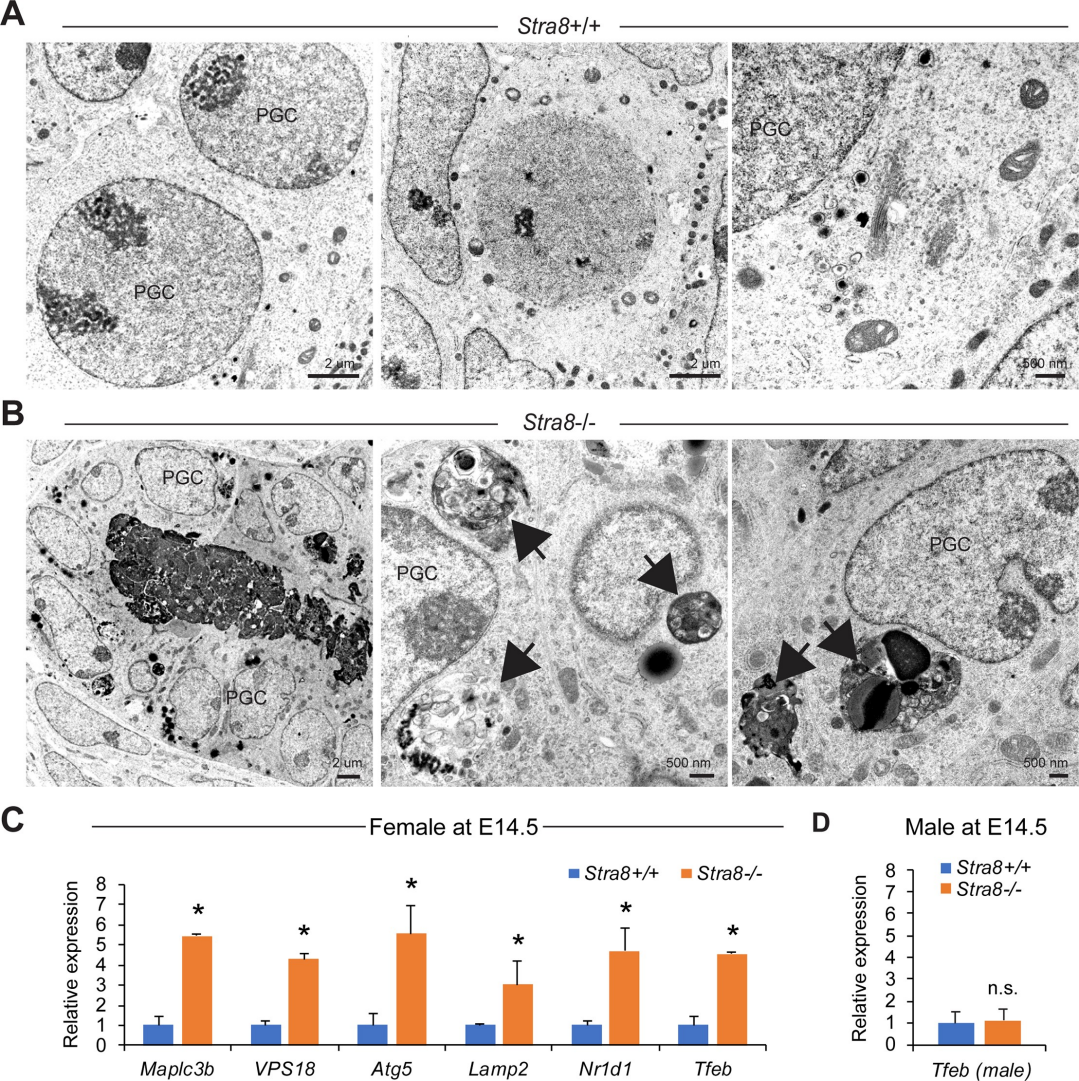
Loss of STRA8 results in aberrant autophagosome formation and upregulation of autophagy-lysosome gene expression in fetal ovarian germ cells during the developmental window of meiotic initiation. (**A, B**) Transmission electron microscopy images of ovarian cross sections from wild-type (**A**) and *Stra8*-deficient (**B**) mice at embryonic day 14.5 (E14.5) that show primordial germ cells (PGC). Arrows indicate autophagosomes. (**C, D**) qRT-PCR analysis of autophagy and lysosome gene expression in wild-type and *Stra8*-deficient fetal ovaries (**C**) and testes (**D**) at E14.5 normalized to germ cell content by *Mvh* levels. Data represent mean ± SD; n = 3 embryos per group. *P < 0.05 (Student’s *t* test).

Despite dramatic sexual dimorphism in mammalian meiosis [45, 46], STRA8 appears to exhibit a comparable role in inducing both male and female meiosis [7, 8]. Thus, to examine whether STRA8 adopts similar mechanism of autophagy suppression in inducing meiosis in females, we investigated female meiosis, which occurs during embryonic day 13.5 (E13.5) to E16.5. Consistent with the observations in postnatal *Stra8*-deficient testes, autophagosome structures were frequently identified in germ cells of *Stra8*-deficient E14.5 ovaries (17 autophagosomes observed in 119 germ cells from 2 ovaries). In contrast, similar structures were not observed in age-matched wild-type fetal ovaries (158 germ cells from 2 wild-type fetal ovaries examined) (**Fig 10A and 10B**). Moreover, autophagy-lysosome genes as well as the STRA8 target gene, *Nr1d1*, were significantly upregulated in *Stra8*-deficient ovaries when compared to wild-type ovaries at the same developmental stage (**Fig 10C**). However, similar changes were not observed in embryonic testes at this age, because at this age meiosis is still inactive and STRA8 is absent until after birth (**Fig 10D**). Taken together, the results suggest that, similar to its role in male germ cell meiosis, STRA8 functions as a suppressor of autophagy in female meiosis.

## Discussion

**Fig 11:**
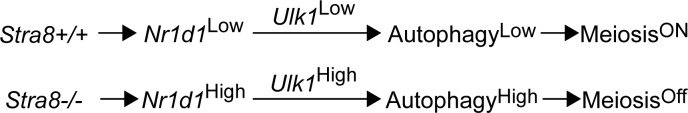
Schematic model of STRA8-mediated meiosis initiation by suppressing autophagy through NR1D1-ULK1 axis.

Despite being an essential gatekeeper of meiotic initiation in mammalian germ cells, the molecular function of STRA8 has remained elusive to date. Here, by using mouse spermatogenesis as a model together with *in vitro* biochemical assays for autophagy, we report that STRA8 acts as a suppressor of autophagy by repressing *Nr1d1* expression and consequently, inhibiting *Ulk1* expression (**Fig 11**). Our data suggest a novel link between suppression of autophagy and meiotic initiation during mammalian germ cell development.

### Molecular characterization of STRA8

*Stra8* was first reported as an essential gatekeeper of meiotic initiation in 2006 [7]. To our knowledge, the first study attempted to characterize the molecular function of STRA8 reports that STRA8 shuttles between nucleus and cytoplasm, but is mostly nuclear in freshly isolated germ cells [4]. This agrees with a study using immunostaining in PFA-fixed testicular sections, which localized STRA8 predominantly to the nucleus of meiosis-entering preleptotene spermatocytes [6]. In addition, protein-DNA cross-link studies showed STRA8 had DNA binding activity [4]. Moreover, STRA8 displays transcriptional activity when fused to a GAL4-DNA binding domain, [4]. Although these *in vitro* assays suggest STRA8 as a transcription factor, the targets of STRA8 and their molecular consequences have yet to be identified.

To characterize the meiotic gene program regulated by STRA8 *in vivo*, Soh and colleagues conducted RNA-sequencing analysis in wild-type and *Stra8*-deficient fetal ovaries at E14.5, when ovarian germ cells enter meiosis [47]. However, only meiotic genes were not selected for investigation by Soh and colleagues. Interestingly, examination of this earlier RNA-sequencing result revealed notable similarities to the data reported herein, namely, the autophagy-lysosome genes that exhibit significant upregulation in both *Stra8*-deficient testes at 10 d.p.p. and *Stra8*-deficient fetal ovaries at E14.5 in our study (**Fig 4 and Fig 10C**, respectively), including *Ulk1*, *Atg5*, *Map1lc3b*, *Lamp2*, *Vps18*, and *Tfeb*, also exhibited tendency of being upregulated in *Stra8*-deficient fetal ovaries at E14.5 in the RNA-sequencing result of Soh and colleagues. Moreover, except for *Map1lc3a*, the autophagy-lysosome genes that were not upregulated in *Stra8*-deficient testes at 10 d.p.p. in our study (**Fig 4**), i.e., *Pik3c3*, *Atg7*, *Vps11*, *Uvrag*, *Lamp1*, and *Becn1*, were also not upregulated in *Stra8*-deficient fetal ovaries at E14.5 in the RNA-sequencing result of Soh and colleagues. Thus, changes in autophagy-lysosome gene expression in *Stra8*-deficient fetal ovaries at E14.5 detected by Soh and colleagues using RNA-sequencing align with the data presented in the current study. These data together suggest that STRA8 inhibits the expression of a selective autophagy-lysosome gene program, thereby suggesting STRA8 functions as s suppressor of autophagy.

A more recent study by Shen and colleagues is focused on characterizing a potential role for STRA8 in preventing apoptosis of germ cells [48]. Generally speaking, autophagy is associated with a response to stress to prevent cells death; however, excessive autophagy leads to apoptosis [49]. *Stra8*-deficient germ cells exhibited profound activation of autophagy (**Figs 1–4**) which may represent a condition of uncontrolled autophagy activation that leads to their germ cell apoptosis [8].

### Autophagy in meiosis

To date, the roles of autophagy regulation in meiosis remains unclear. In budding yeast (*S*. *cerevisiae*), autophagy participates in the early phase of meiosis and is switched off upon meiotic division [50]. In fission yeast (*S*. *pombe*), however, autophagy is thought to be required for chromosome segregation during meiotic division [51]. Thus, these observations underscore the concept that mechanisms of meiotic initiation could differ in model organisms [2].

Our observation that autophagy is being actively suppressed by STRA8 during meiotic initiation in mouse spermatogenesis is in accordance with a recent study of autophagy in rat spermatogenesis, in which autophagy was found only to be activated during late meiotic spermatocytes but not in spermatogonia and early spermatocytes [19]. This is in line with the finding in yeast, in which autophagy is required for proper meiotic chromosome segregation [51]. Together, our findings suggest that activation of autophagy is specifically prevented by STRA8 during meiotic initiation in mammalian germ cell development. It should be noted that *Stra8*-deficient germ cells, when rescued by inhibiting NR1D1 antagonist SR8278, do not progress beyond leptotene stage (**S10 Fig**), suggesting that STRA8 has additional role(s) beyond the initiation of meiosis through NR1D1-independent mechanisms. For instance, based on our RNA-seq analysis, 15 out of 22 STRA8-regulated genes of are noncoding RNAs (**Fig 7A**). Given that non-coding RNAs play an essential role in meiosis prophase and homologous recombination [35], it is possible that STRA8 regulates this stage of meiosis through noncoding RNAs.

### NR1D1 in transcriptional autophagy regulation

We observed that STRA8-deficiency resulted in a significantly autophagy-lysosome gene expression in testes; reciprocally, these autophagy-lysosome genes were downregulated in cultured cells with stable STRA8 expression. Based on these data and a past study that indicated STRA8 could bind to DNA and display transcriptional activity [4], we had expected that STRA8, like some other transcriptional regulators of autophagy, such as TFEB [13] or FXR [52], could potentially regulate a wide spectrum of autophagy-lysosome genes directly. However, no autophagy-lysosome genes were identified as direct targets of STRA8 by transient ectopic expression of STRA8 in our *in vitro* RNA-sequencing analysis under basal condition. Instead, *Nr1d1* was identified as a target repressed by STRA8 transcriptionally. To date, three studies have reported a role of NR1D1 in either inducing or inhibiting autophagy, all involving transcriptional regulation of *Ulk1* [29–31]. While Woldt and colleagues reported that NR1D1-deficiency in murine muscle leads to upregulation of autophagy [30], Chandra and colleagues reported that *Nr1d1*-knock down leads to reduced autophagy and downregulation of *Ulk1* gene expression in human macrophage [29]. Interestingly, whether NR1D1 is an autophagy inducer or inhibitor remains unclear in the study reported by Huang and colleagues [31]. Thus, these studies point out a critical regulatory role of NR1D1 in autophagy through modulating autophagy-lysosome gene expression. Herein, the data show that genetic loss of NR1D1 prevented the upregulated *Ulk1* expression normally observed in *Stra8*-knockout testes (**Fig 8D**), supporting a role for NR1D1 as an inducer of autophagy by activating autophagy-lysosome gene expression during germ cell development. It is currently unclear why there is a robust activation of autophagy that is precisely counteracted by STRA8-mediated suppression of autophagy during the transition to meiosis. One possibility is that the activation of autophagy is simply a response to cellular stress. A second, and more intriguing, possibility is that autophagy induction also plays an essential role in meiotic initiation and is an area that warrants further investigation.

### Induction of DSBs as a potential role for autophagy suppression in meiotic initiation

Based primarily on models of somatic tumorigenesis, genetic disruption of critical autophagy factors can induce the formation of DNA DSBs upon metabolic stress [14, 15]. In germ cells, DSBs are required to initiate meiosis and permit the exchange of genetic information between maternal and paternal chromosomes through homologous recombination [53]. *Stra8* activation is required for meiotic DSB formation [7, 8]. Thus, by characterizing STRA8 as a suppressor of autophagy, our work suggests autophagy suppression as a possible mechanism adopted by germ cells to form meiotic DSBs. The mechanisms underlying autophagy inhibition-induced DSB formation is currently unclear. Several studies show that autophagy-deficiency causes DNA damage through accumulation of autophagic substrate, p62 [54–56]. However, *p62*-deficient mice are reportedly to be fertile [57], suggesting that p62 accumulation alone is dispensable for meiosis. In addition, loss of autophagy has been shown to affect DNA damage repair machineries, such as HIPα (a molecule essential for chromosome condensation) [16] and Chk1 (a molecular regulator of DNA damage repair by homologous recombination) [58]. Thus, a role for autophagy suppression in DSB formation during meiotic initiation remains to be determined.

### Implication for *in vitro* meiotic induction in germ cell production

Mounting efforts have been directed to derive functional haploid gametes (sperm or oocytes) from spermatogonial stem cells, embryonic stem cells or induced pluripotent stem cells in cultures [59]. However, how to properly induce and sustain meiosis remains to be a major challenge in this field. Our study revealed that germ cells entering meiosis is exposed to an antagonistic pressure of simultaneous autophagy induction and STRA8-mediated autophagy suppression. Thus, manipulating autophagy pathway to mimic this *in vivo* condition may facilitate the induction of meiosis in cultures, thereby advancing the technology of *in vitro* haploid gamete production that may ultimately afford clinical utility in assisted reproduction technology.

## Materials and methods

### Animals and treatments

All genetically modified mice were obtained from Jackson Laboratory: *Stra8*-deficient mice (Stock number: 023805), RFP-GFP-LC3 transgenic mice (Stock number: 027139), and *Nr1d1*-deficient mice (Stock number: 018447). For chloroquine treatment, mice were injected intraperitoneally with chloroquine dissolved in PBS at 100 mg/kg body weight daily. For SR8278 treatment, mice at 7 days of age were injected intraperitoneally with vehicle (5% DMSO, 10% Cremephol, and 85% PBS) or SR8278 at 100 mg/kg body weight daily for 3 days. Postnatal testes were dissected from male mice after euthanasia. For timed pregnancy, females at estrus stage were caged with males overnight, and the presence of vaginal plug was examined the following morning and the midday was defined as E0.5. Pregnant females were euthanized when embryos reached E14.5. Embryos were collected from uterine horns before gonad isolation under a binocular dissecting microscope.

### Ethics statement

All procedures and care of animals were carried out according to the Massachusetts General Hospital (MGH) and the University of Kansas Medical Center (KUMC) Institutional Animal Care and Use Committee (IACUC) under IACUC protocol number 2012N000015 and 2018–2461, respectively. Euthanasia was performed by CO_2_ inhalation followed by cervical dislocation (MGH) and decapitation (KUMC).

### Electron microscope

Tissue specimens were fixed in 2.5% glutaraldehyde in 0.1M sodium phosphate buffer (pH 7.4, Electron Microscopy Sciences, Hatfield, PA), then rinsed several times in 0.1M sodium cacodylate buffer. Specimens were post-fixed in 1.0% osmium tetroxide in cacodylate buffer for 1 hour at room temperature and rinsed several times in cacodylate buffer. Samples were then dehydrated through a graded series of ethanols to 100% and dehydrated briefly in 100% propylene oxide. Samples were pre-infiltrated 2 hours in a 2:1 mix of propylene oxide and Eponate resin (Ted Pella, Redding, CA), then transferred into a 1:1 mix of propylene oxide and Eponate resin and allowed to infiltrate overnight on a gentle rotator. The following day, specimens were infiltrated with fresh 100% Eponate resin for several hours, embedded in flat molds with fresh 100% Eponate and allowed to polymerize 24–48 hours at 60°C. Thin (70nm) sections were cut using a Leica EM UC7 ultramicrotome, collected onto formvar-coated grids, stained with uranyl acetate and Reynold's lead citrate and examined in a JEOL JEM 1011 at MGH or The JEOL JEM-1400 at KUMC transmission electron microscope at 80 kV. Images were collected using an AMT digital imaging system (Advanced Microscopy Techniques, Danvers, MA).

### Plasmids

Human STRA8 expression plasmid was obtained from Origene (RC213536). To tag STRA8 with GFP, STRA8 cDNA was inserted into the pEGFP-N1 vector (Clontech). Mutant STRA8 plasmids were generated using Q5 mutagenesis kit (New England Biolabs). All plasmids were sequenced to confirm fidelity prior to use.

### Cell culture

F9 embryonic carcinoma cells (ATCC CRL-1720; American Type Culture Collection) and 293T were cultured at 37°C in a humidified atmosphere of 5% CO_2_-95% air in DMEM containing 4.5 g/l glucose and supplemented with 10% FBS (Invitrogen), 2 mM L-glutamine, 50 μg/ml penicillin and 50 μg/ml streptomycin. F9 cells were cultured on 0.1% gelatin-coated tissue culture plates. Cells were transfected using Lipofectamine 2000 (Invitrogen) and then stably selected by G418 (Invitrogen) for 2 to 3 weeks, before STRA8-expressing cells (GFP-positive) were identified and isolated by flow cytometry. F9 cells expressing GFP alone were used as control. For LC3 staining, cells were fixed in ice-cold methanol for 2 minutes before immunostaining with antibody against LC3 (12741, Cell Signaling Technology). Images were captured by using a ZEISS LSM 800 confocal microscopy with Airyscan. To induce autophagy, control or Stra8-expressing cells were treated with rapamycin (0.1 mM), metformin (2 mM), or Earle's balanced salt solution (EBSS) for 2 hours before cell lysates were collected. To monitor the effects of blocking autophagosome maturation, control or STRA8-expressing cells were treated with chloroquine (20 μM) for 2 hours before cell lysates were collected. Hela cells carrying mRFP-GFP-LC3 were transfected by plasmid expressing empty pCMV6 vector or STRA8 (not tagged with GFP). Then, cells were fixed by 4% paraformaldehyde for 10 minutes at room temperature before observation under fluorescence microscope.

### Immunostaining

Testes were fixed in 4% paraformaldehyde, embedded in paraffin, and sectioned for analysis. Antibodies used include: p62 (ab56416, abcam; 1:2000 dilution for immunofluorescence and 1:10,000 dilution for immunohistochemistry), γ-H2AX 05–636, Millipore; 1:1,000 dilution), NR1D1 (sc-100910, Santa Cruz Biotechnology; 1:500 dilution), SYCP3 (sc-74569, Santa Cruz Biotechnology; 1:500 dilution). For immunofluorescence, detection was performed using Alexa fluor 546-conjugated goat anti-rabbit antibody and Alexa fluor 488-conjugated goat anti-mouse secondary antibodies. For immunohistochemistry, detection was performed using goat anti-mouse or goat anti-rabbit as secondary antibody for horseradish peroxidase-based DAB detection (DAKO). Images were captured using a Nikon ECLIPSE TE2000-S microscope and were analyzed by Image J software (National Institutes of Health).

### Imaging of the RFP-GFP-LC3 reporter

Dissected seminiferous tubules from wild-type and *Stra8*-deficient testes carrying the RFP-GFP-LC3 reporter were fixed in 4% paraformaldehyde on ice for an hour. Tissue is washed with PBS to remove PFA. For whole-mount imaging, tissues of seminiferous tubules were mounted directly on glass slides in PBS for confocal microscopy by using a Nikon A1R microscope. For imaging on testicular cross sections, testes were cryosectioned. Then sections were washed with warm PBS to remove gelatin and were imaged under Nikon ECLIPSE TE2000-S microscope. No staining was performed under both conditions. Images from wild-type and *Stra8*-deficient samples mounted on the same slide were captured and processed in parallel using identical settings.

### Western blot

Total protein was isolated in RIPA buffer supplemented with 1 mM PMSF (Sigma) and protease inhibitor cocktail (Sigma P8340). Lysates were cleared by centrifugation at 14,000 X *g* for 10 min at 4°C, and protein concentrations in supernatants were determined (DC protein assay; BioRad). Equal amount of protein from each sample was mixed with LDS sample buffer (Invitrogen) plus sample reducing agent (Invitrogen), and denatured for 10 min at 70°C. Proteins were resolved in Bis-Tris gels (Thermo Fisher), and transferred to PDVF membranes. Blots were probed with antibodies against LC3A/B (12741, Cell Signaling Technology; 1:1,000 dilution), p62 (ab56416, Abcam; 1:20,000 dilution), GFP (sc-9996, Santa Cruz Biotechnology; 1:1,000 dilution), or pan-actin (MS-1295, Thermo Fischer; 1:1,000 dilution), washed and reacted with horseradish peroxidase-conjugated goat anti-rabbit or anti-mouse IgG (BioRad). Detection was performed with the Clarity ECL Western Blotting Substrate (BioRad).

### Chromatin immunoprecipitation (ChIP)

293T cells were transfected with plasmid expressing GFP-tagged wild-type and mutant STRA8. 24 hours later, cell lysates were processed using the EZ-ChIP kit (Millipore, Temecula, CA) along with a rabbit polyclonal anti-GFP antibody (ab290; Abcam) for immunoprecipitation. Normal rabbit IgG was used as a negative control. Precipitated soluble chromatin was analyzed by PCR using primer sets to target E-box: forward, 5’-CCC TCC CCG GCT TCT CTC TCT CC-3’, reverse, 5’-GCA AAC CTT GCA AAC GTG AGG GC-3’; and to target exon 8: forward, 5’-CCG GAC CTG CGG ACC CTG AAC AA-3’, reverse, 5’-TCT GTA CAA GGG GGC AGC GGC AGA-3’.

To characterize NR1D1 binding to the *Ulk1* promoter, testicular lysates were processed using the EZ-ChIP kit (Millipore, Temecula, CA) along with a rabbit polyclonal anti-NR1D1 antibody (#13418; Cell Signaling Technology) for immunoprecipitation. Normal rabbit IgG was used as a negative control. Precipitated soluble chromatin was analyzed by PCR using primer sets to target ROREs: primer set 1, forward, 5’-AAT GGG TAT GTG CGA CAA CA-3’, reverse, 5’-TGT CAT TTG GGG AGG GGT AT-3’; primer set 2, forward, 5’-TGC CAA GTT TGA CAA CCT GA-3’, reverse, 5’-CTG TAT GTG GGG ACG GAG AC-3’; primer set 3, forward, 5’-GCA CCT GCC TTT AAT TCC AA-3’, reverse, 5’-CGA CTG GTC TCG AAC TTG CT-3’.

### RNA-sequencing

MCF-7 cells were transiently transfected with control (pCMV6) or STRA8 plasmid. Cells were collected after 24 hours. Total RNA was isolated using the RNeasy Mini Kit (Qiagen). Following rRNA depletion using RiboZero kit (Epicentre/Illumina), RNA-Seq libraries were constructed using NEBNext Ultra Directional RNA library prep kit for Illumina (New England Biolabs) and sequenced on Illumina HiSeq2500 instrument, resulting in approximately 25 million reads per sample on average. STAR aligner was used to map sequencing reads to transcripts in hg19 reference genome. Read counts for individual transcripts were produced with HTSeq-count, followed by the estimation of expression values and detection of differentially expressed transcripts using EdgeR. Principle component analysis (PCA) was performed on the union of differentially expressed transcripts in all samples.

### In situ hybridization procedures (ISH)

In situ hybridization (ISH) has been described previously [60]. Antisense *Nr1d1* probe (825 bp) and *Ulk1* probe (843 bp) were amplified from cDNA prepared from juvenile testes, and labeled with digoxigenin (Roche Diagnostics).

### Isolation of c-Kit-negative and c-Kit-positive spermatogenic cells

Testes were collected from age-matched wild-type and *Stra8*-deficient mice. Testicular cells were dissociated by two-step enzymatic digestion, followed by staining with PE-conjugated rat anti-human integrin α6 antibody (BD Pharmingen, clone GoH3) and APC-conjugated rat anti-mouse c-Kit antibody (BD Pharmingen, clone 2B8) as previously described [32]. 40,000–50,000 cells from each population were sorted directly into lysis buffer in NucleoSpin RNA XS kit (Takara) for subsequent RNA isolation.

### Gene expression analysis

Equal amount of total RNA from each sample was reverse transcribed by using SuperScript III from Invitrogen. quantitative RT-PCR was conducted by using SsoAdvanced Universal SYBR Green Supermix (BioRad). Primer sequences are listed below:

*β-actin*    forward, 5’-CTG CCG CAT CCT CTT CCT C-3’

                    reverse, 5’-GCC ACA GGA TTC CAT ACC CA-3’

*Mvh*    forward, 5’-GCT TCA TCA GAT ATT GGC GAG T-3’

                    reverse, 5’-GCT TGG AAA ACC CTC TGC TT-3’

*Maplc3A*    forward, 5’-CAC ATC CTG GGT AGG TCC TG-3’

                    reverse, 5’-AAT GAC AAA CCC CAC AGA GC-3’

*Maplc3B*    forward, 5’-CGG CTT CCT GTA CAT GGT TT-3’

                    reverse, 5’-AAC CAT TGG CTT TGT TGG AG-3’

*Atg12*    forward, 5’-TCC CCG GAA CGA GGA ACT C-3’

                    reverse, 5’-TTC GCT CCA CAG CCC ATT TC-3’

*Atg5*    forward, 5’-TGT GCT TCG AGA TGT GTG GTT-3’

                    reverse, 5’-GTC AAA TAG CTG ACT CTT GGC AA-3’

*Atg7*    forward, 5’-GTT CGC CCC CTT TAA TAG TGC-3’

                    reverse, 5’-TGA ACT CCA ACG TCA AGC GG-3’

*Pik3c3*    forward, 5’-CCT GGA CAT CAA CGT GCA G-3’

                    reverse, 5’-TGT CTC TTG GTA TAG CCC AGA AA-3’

*Tfeb*    forward, 5’-CCA CCC CAG CCA TCA ACA C-3’

                    reverse, 5’-CAG ACA GAT ACT CCC GAA CCT T-3’

*Ulk1*    forward, 5’-AAG TTC GAG TTC TCT CGC AAG-3’

                    reverse, 5’-CGA TGT TTT CGT GCT TTA GTT CC-3’

*UVRAG*    forward, 5’-ACA TCG CTG CTC GGA ACA TT-3’

                    reverse, 5’-CTC CAC GTC GGA TTC AAG GAA-3’

*Vps11*    forward, 5’-AAA AGA GAG ACG GTG GCA ATC-3’

                    reverse, 5’-AGC CCA GTA ACG GGA TAG TTG-3’

*Vps18*    forward, 5’-ACG AGG ACT CAT TGT CCC G-3’

                    reverse, 5’-CAT ACC CAG AAT GGG GGA TGC-3’

*Lamp1*    forward, 5’-CAG CAC TCT TTG AGG TGA AAA AC-3’

                    reverse, 5’-ACG ATC TGA GAA CCA TTC GCA-3’

*Lamp2*    forward, 5’-TGT ATT TGG CTA ATG GCT CAG C-3’

                    reverse, 5’-TAT GGG CAC AAG GAA GTT GTC-3’

*Sqstem1*    forward, 5’-AGG ATG GGG ACT TGG TTG C-3’

                    reverse, 5’-TCA CAG ATC ACA TTG GGG TGC-3’

*Beclin1*    forward, 5’-ATG GAG GGG TCT AAG GCG TC-3’

                    reverse, 5’-TCC TCT CCT GAG TTA GCC TCT-3’

*Nr1d1*    forward, 5’-ATG CCC ATG ACA AGT TAG GC-3’

                    reverse, 5’-CGG TGT GGA GTT GTA GCT GA-3’

### Data presentation and analysis

All experiments were replicated at least three times independently. Different mice, tissues or cells were used during each experimental replicate. Animal assignment to each experimental group was made randomly. Quantitative data from the experimental replicates were pooled and are presented as the mean ± SEM or mean ± SD as indicated in the figure legend. Compiled data were analyzed by Student’s *t*-test.

## Supporting information

S1 Fig Direct fluorescence microscopy of whole-mount seminiferous tubules from chloroquine (CQ)-treated wild-type and *Stra8*-deficient testes. Images are merged of GFP and RFP channels. Note the accumulation of autophagosomes (AP; GFP-positive and RFP-positive, yellow) in CQ-treated *Stra8*-deficient testes indicated by arrows. Numbers of APs were quantified in randomly selected areas of tubules dissected from 3 different age-matched wild-type and *Stra8*-deficient juvenile mice treated with CQ for 3 days at 100 mg/kg.
(TIF)

S2 Fig Immunohistochemical staining of p62 in testes from vehicle (PBS)- and chloroquine (CQ)-treated wild-type testes.
(TIF)

S3 Fig (**A**) qRT-PCR analysis of *Mvh* in wild-type and *Stra8*-deficient testes at 10 d.p.p. normalized to *β-actin*. Data represent mean ± SD; n = 5 mice per group. (**B**) Immunofluorescence staining of MVH in wild-type and Stra8-deficient testes at 10 d.p.p.
(TIF)

S4 Fig (**A**) Schematic of human STRA8 tagged with GFP at C-terminus. (**B**) Western blot analysis of STRA8 (tagged with GFP) and GFP expression in transfected 293T cell lysate with respective plasmid detected by GFP antibody. (**C**) Flow cytometric analysis of sorted F9 cells stably expressing GFP or STRA8 (tagged with GFP). Please note that cells stably expressing STRA8 show a quick downregulation of STRA8 expression over days as detected by GFP levels in FACS. Therefore, to ensure conducting experiments with consistent levels of STRA8 expression, F9 cells were routinely sorted by FACS based on GFP intensity that indicates ectopic STRA8 expression and then propagated for downstream analysis.
(TIF)

S5 Fig bHLH domain of STRA8 is required to suppress autophagy. (**A**) Schematic of wild-type STRA8 (STRA8_WT) and its mutants (mNLS, mHelix). (**B**) Cellular localization of GFP, STRA8_WT, mNLS and mHelix (all tagged with GFP) in F9 cells. (**C**, **D**) Western blot analyses of F9 cell lysates from STRA8_WT, mNLS and mHelix with indicated treatments and antibodies. Please note that F9 cells stably expressing comparable levels of STRA8_WT, mNLS and mHelix were sorted by FACS based on GFP intensity before conducting these experiments.
(TIF)

S6 Fig (**A**) Quantitative RT-PCR analysis of *Nr1d1* expression relative to *β-actin* in F9 cells stably expressing GFP (Ctrl), STRA8_WT, mNLS, and mHelix. Data represent mean ± SD; n = 3 independent experiments; * *P* < 0.05 (Student’s *t* test). (**B**) Quantitative RT-PCR analysis of *Nr1d1* expression relative to *β-actin* in MCF-7 cells and 293T cells after transient transfection of pcDNA3.1 (vector) and STRA8. Data represent mean ± SD; n = 3 independent experiments; * *P* < 0.05 (Student’s *t* test). (**C**) qRT-PCR analysis of *Nr1d1* in wild-type and *Stra8*-deficient testes at 10 d.p.p. normalized to *β-actin*. Data represent mean ± SD; n = 5 mice per group.
(TIF)

S7 Fig (**A**) Representative flow profile of dissociated wild-type and *Stra8*-deficient testicular cells stained with APC-conjugated c-Kit and PE-conjugated integrin α6. (**B**) quantification of the percentage of c-Kit-negative integrin α6-high and c-Kit-positive integrin α6-low populations in wild-type and *Stra8*-deficient testes analyzed by FACS in panel (**A**). Graphs represent mean value ± s.e.m. n = 3 mice per group. *P < 0.05. Both undifferentiated and differentiating spermatogonia exhibited expansion in cellular population in *Stra8*-deficient testes, probably due to blocked meiotic initiation.
(TIF)

S8 Fig Quantitative RT-PCR analysis of *Spo11*, *Dmc1*, and *Sycp3* relative to *β-actin* in wild-type and *Stra8*-deficient testes at 10 d.p.p.. Data represent mean ± SD; n = 5 mice per group; *P < 0.05 (Student’s t test).
(TIF)

S9 Fig (**A**) Low power magnification view of *Stra8*^-/-^;*Nr1d1*^+/+^ and *Stra8*^-/-^;*Nr1d1*^-/-^ testes at 10 d.p.p.. Red asterisks in *Stra8*^-/-^;*Nr1d1*^-/-^ testes indicate tubules containing germ cells with condensed chromosomes. Image in lower left corner shows enlargement of areas in red boxes. Arrow heads show germ cells with condensed chromosomes found in *Stra8*^-/-^;*Nr1d1*^-/-^ testes. (**B and D**) Quantitative RT-PCR analysis of *Spo11*, *Dmc1*, and *Sycp3* expression normalized to *β-actin* in testes with indicated genotypes and treatments. Data are mean ± SD; n = 3–5 mice per group. (**C** and **E**) Dual immunofluorescence staining of γ-H2AX and SYCP3 in testes with indicated genotypes and treatments.
(TIF)

S10 Fig *Stra8*-deficient testicular germ cells rescued from meiotic initiation arrest by SR8278 do not undergo meiotic progression. (**A**) Hematoxylin-eosin staining of testicular sections from age-matched wild-type and *Stra8*-deficient mice 7 days post-SR8278 treatment. Arrows in wild-type testes indicate meiotic spermatocytes. (**B**) Immunofluorescence staining for SYCP1/SYCP3 of testicular sections from wild-type and *Stra8*-deficient mice 7 days post-SR8278 treatment. Enlarged area in wild-type testes show meiotic spermatocytes with synaptonemal complex formation by SYCP3 and SYCP1 (arrows). Enlarged area in *Stra8*-deficient testes 7 days post-SR8278 show speckle pattern of SYCP3 and cytoplasmic SYCP1 (arrow heads).
(TIF)
