# Supplementary figures and images for: Meiotic gatekeeper STRA8 suppresses autophagy by repressing Nr1d1 expression during spermatogenesis in mice
Source: PLoS Genet. 2019 May 6;15(5):e1008084. doi: 10.1371/journal.pgen.1008084 (PMC6502318; doi:10.1371/journal.pgen.1008084)

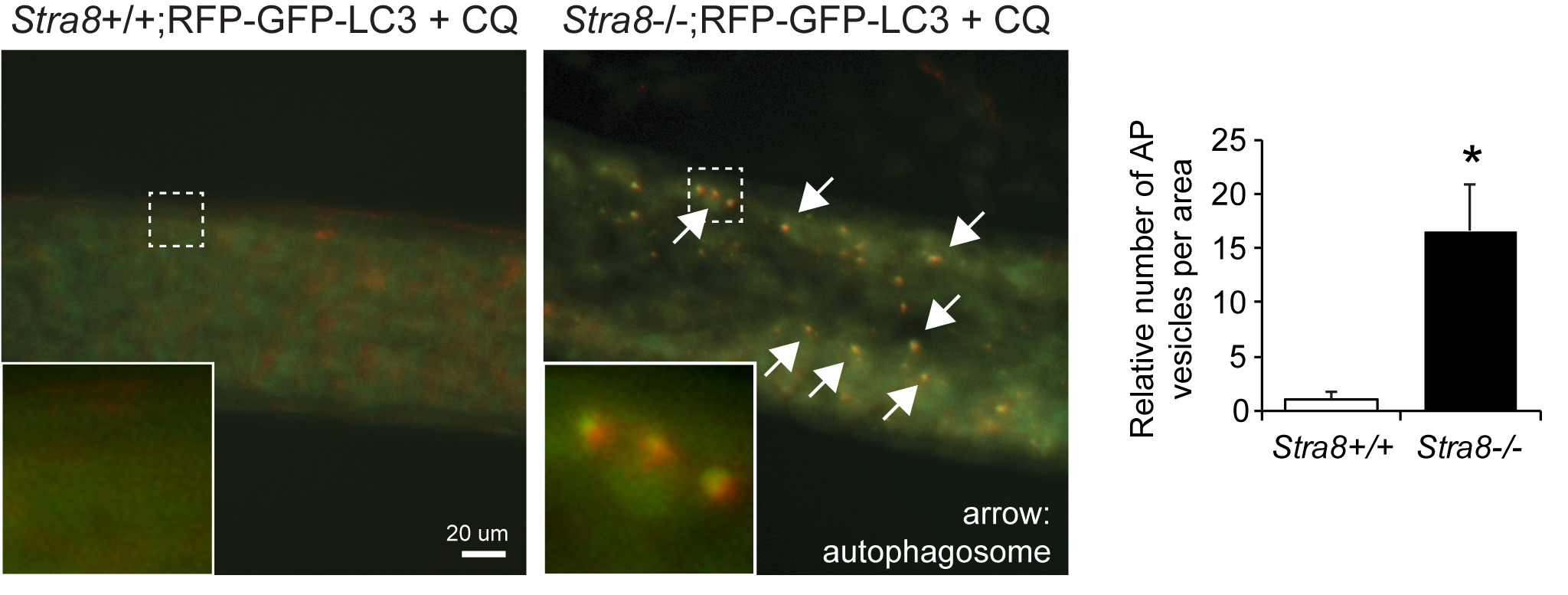

Supplement: S1 Fig — Images are merged of GFP and RFP channels. Note the accumulation of autophagosomes (AP; GFP-positive and RFP-positive, yellow) in CQ-treated Stra8-deficient testes indicated by arrows. Numbers of APs were quantified in randomly selected areas of tubules dissected from 3 different age-matched wild-type and Stra8-deficient juvenile mice treated with CQ for 3 days at 100 mg/kg. (TIF) [file pgen.1008084.s001.tif]

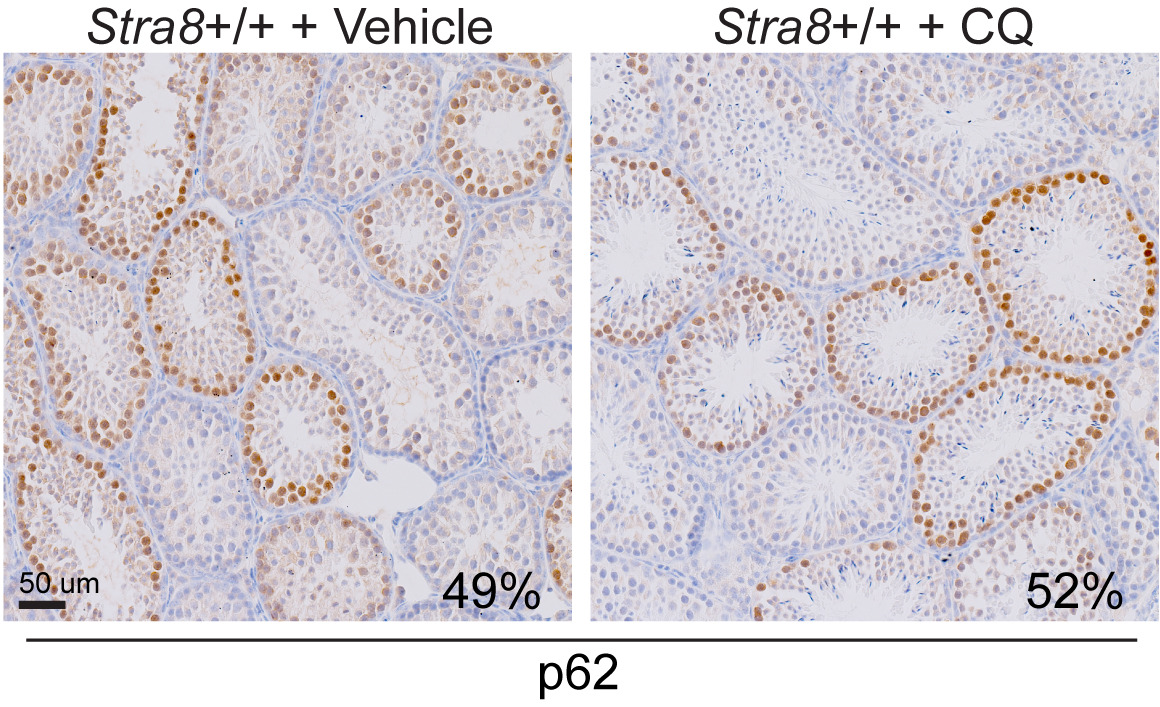

Supplement: S2 Fig — Immunohistochemical staining of p62 in testes from vehicle (PBS)- and chloroquine (CQ)-treated wild-type testes. (TIF) [file pgen.1008084.s002.tif]

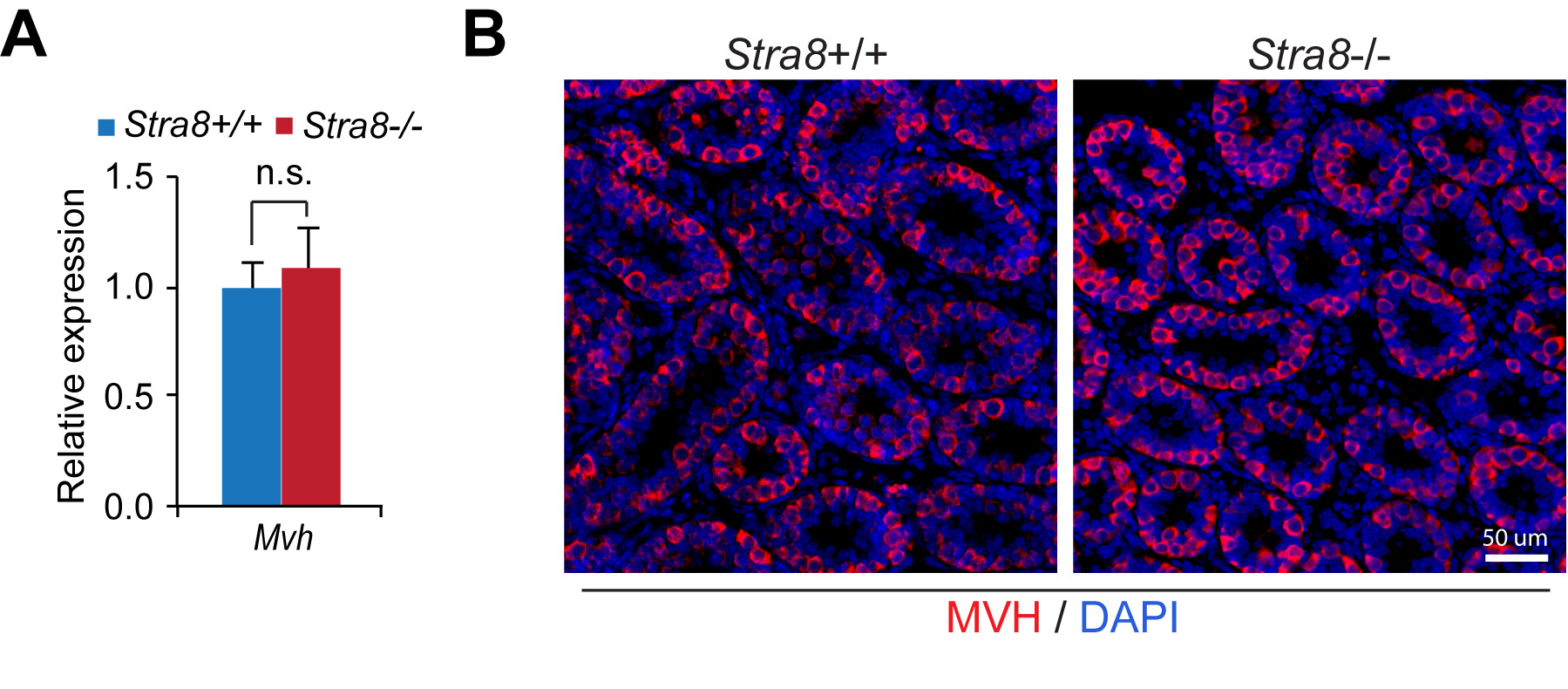

Supplement: S3 Fig — (A) qRT-PCR analysis of Mvh in wild-type and Stra8-deficient testes at 10 d.p.p. normalized to β-actin. Data represent mean ± SD; n = 5 mice per group. (B) Immunofluorescence staining of MVH in wild-type and Stra8-deficient testes at 10 d.p.p. (TIF) [file pgen.1008084.s003.tif]

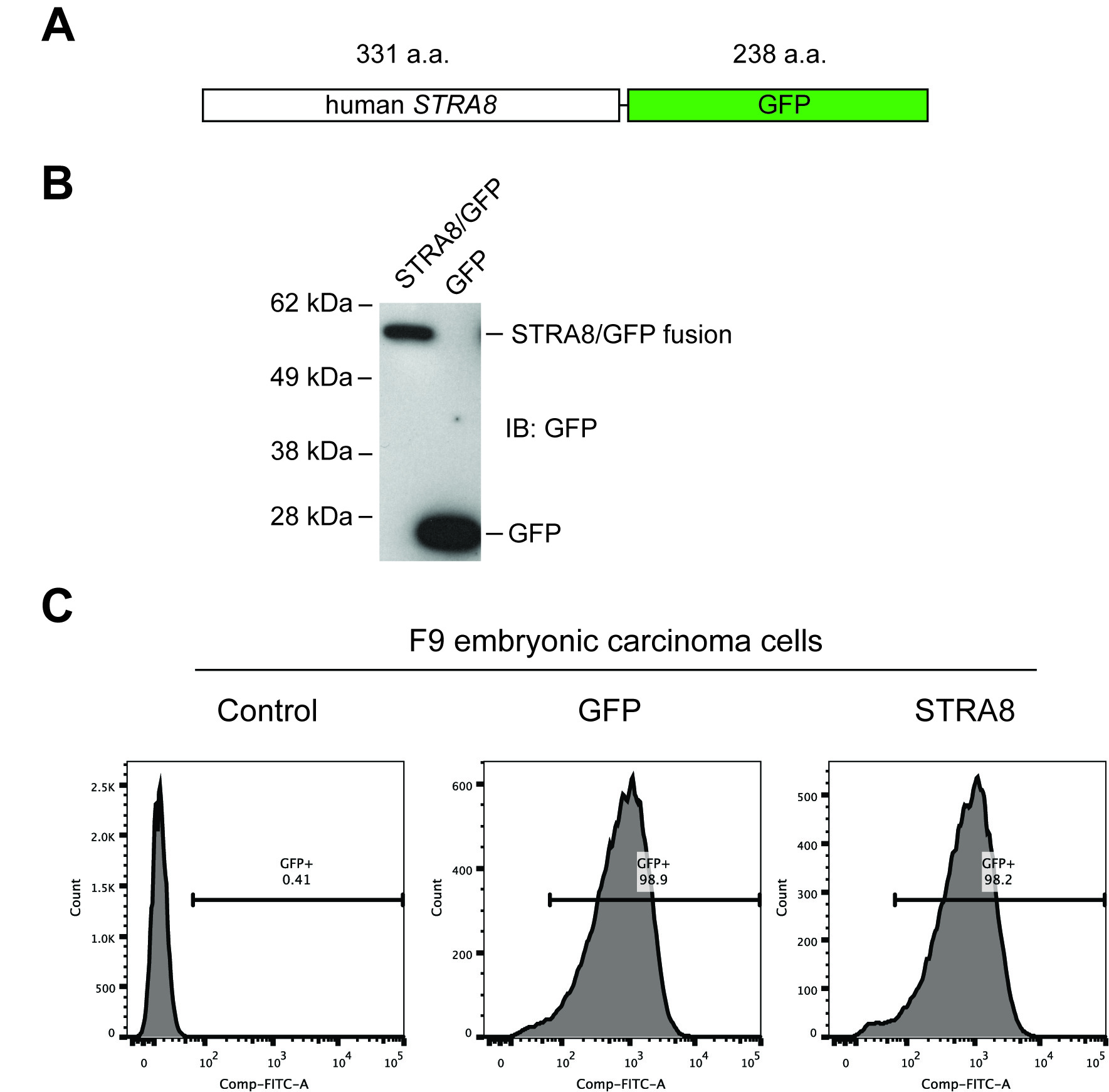

Supplement: S4 Fig — (A) Schematic of human STRA8 tagged with GFP at C-terminus. (B) Western blot analysis of STRA8 (tagged with GFP) and GFP expression in transfected 293T cell lysate with respective plasmid detected by GFP antibody. (C) Flow cytometric analysis of sorted F9 cells stably expressing GFP or STRA8 (tagged with GFP). Please note that cells stably expressing STRA8 show a quick downregulation of STRA8 expression over days as detected by GFP levels in FACS. Therefore, to ensure conducting experiments with consistent levels of STRA8 expression, F9 cells were routinely sorted by FACS based on GFP intensity that indicates ectopic STRA8 expression and then propagated for downstream analysis. (TIF) [file pgen.1008084.s004.tif]

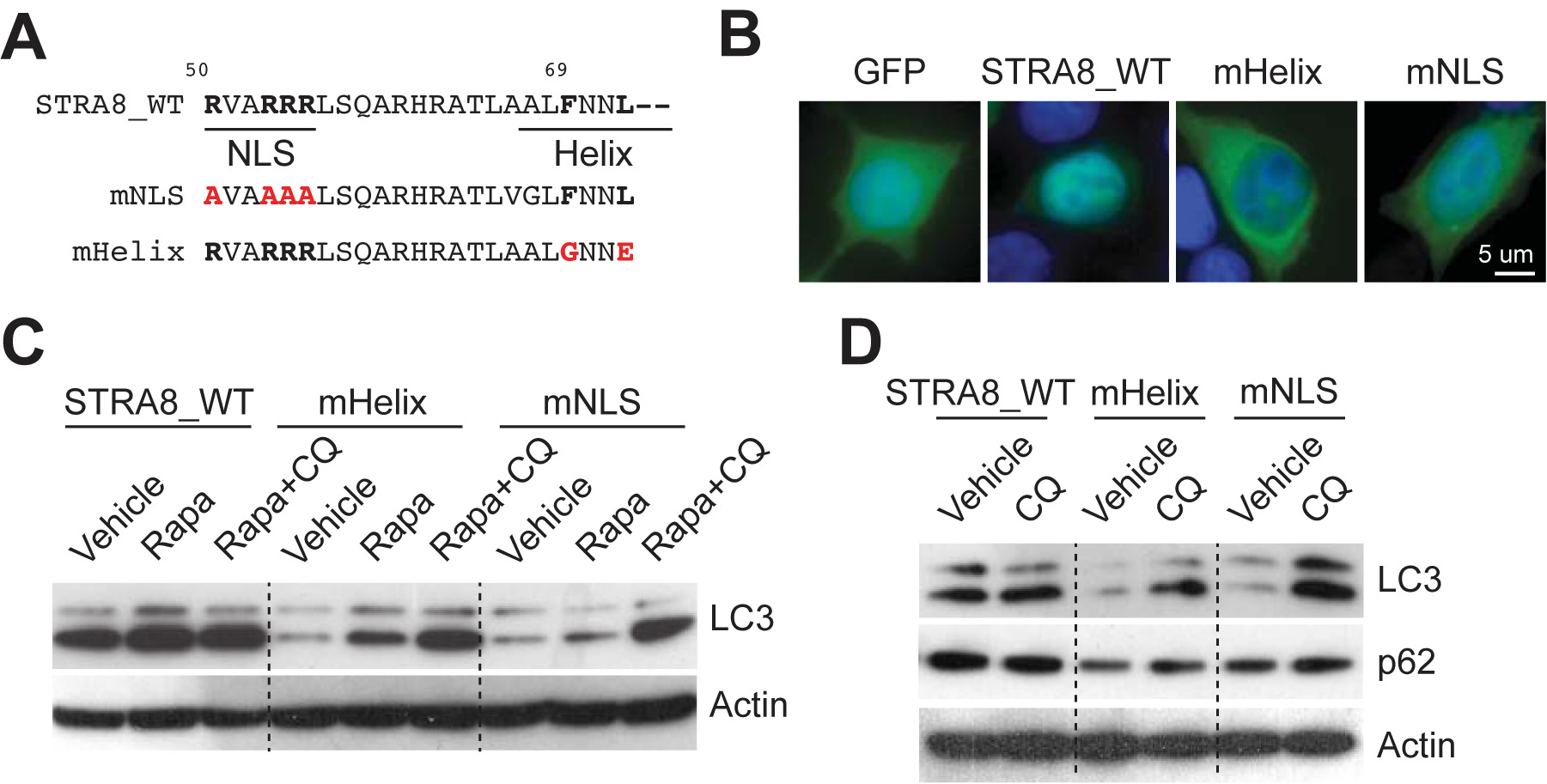

Supplement: S5 Fig — (A) Schematic of wild-type STRA8 (STRA8_WT) and its mutants (mNLS, mHelix). (B) Cellular localization of GFP, STRA8_WT, mNLS and mHelix (all tagged with GFP) in F9 cells. (C, D) Western blot analyses of F9 cell lysates from STRA8_WT, mNLS and mHelix with indicated treatments and antibodies. Please note that F9 cells stably expressing comparable levels of STRA8_WT, mNLS and mHelix were sorted by FACS based on GFP intensity before conducting these experiments. (TIF) [file pgen.1008084.s005.tif]

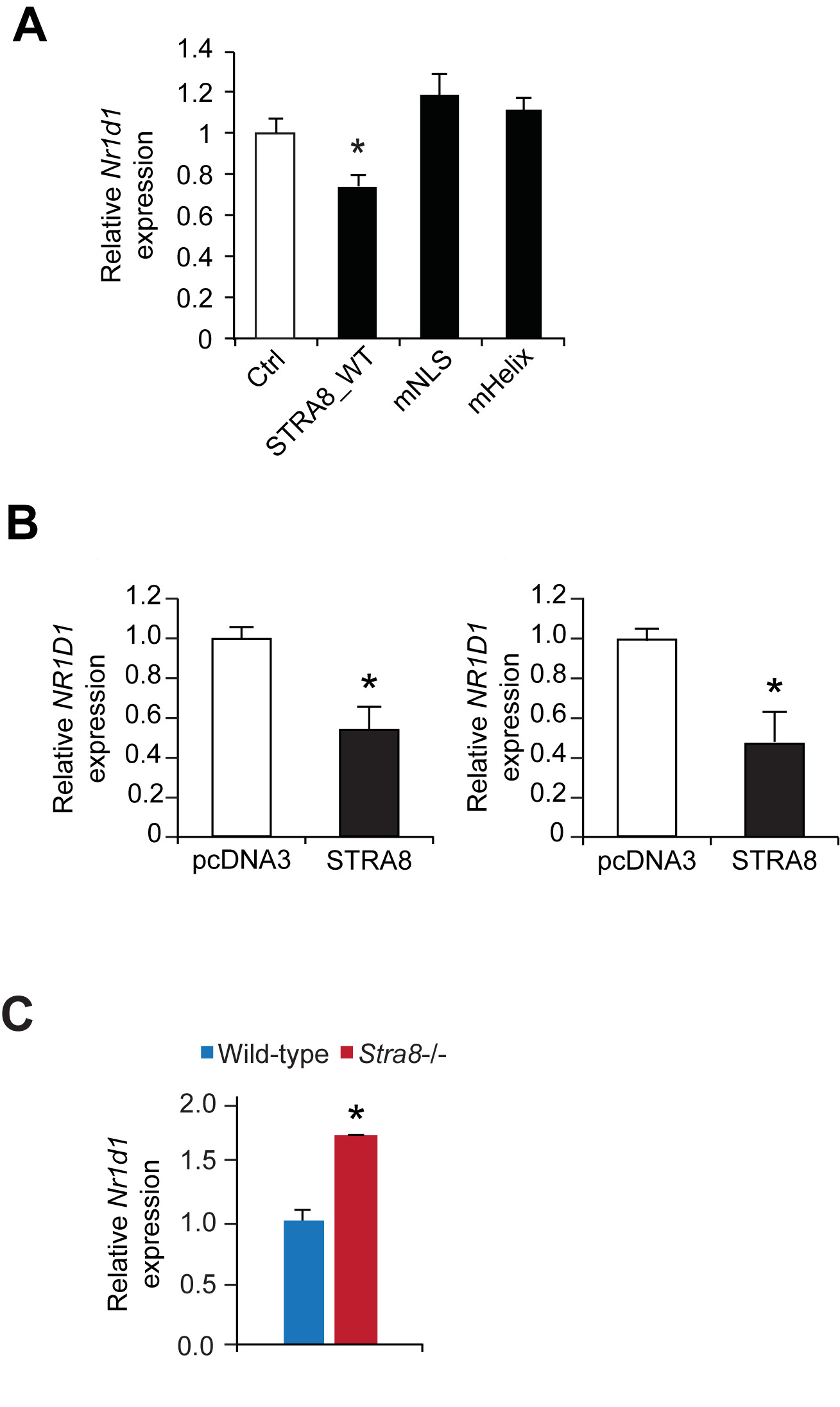

Supplement: S6 Fig — (A) Quantitative RT-PCR analysis of Nr1d1 expression relative to β-actin in F9 cells stably expressing GFP (Ctrl), STRA8_WT, mNLS, and mHelix. Data represent mean ± SD; n = 3 independent experiments; * P < 0.05 (Student’s t test). (B) Quantitative RT-PCR analysis of Nr1d1 expression relative to β-actin in MCF-7 cells and 293T cells after transient transfection of pcDNA3.1 (vector) and STRA8. Data represent mean ± SD; n = 3 independent experiments; * P < 0.05 (Student’s t test). (C) qRT-PCR analysis of Nr1d1 in wild-type and Stra8-deficient testes at 10 d.p.p. normalized to β-actin. Data represent mean ± SD; n = 5 mice per group. (TIF) [file pgen.1008084.s006.tif]

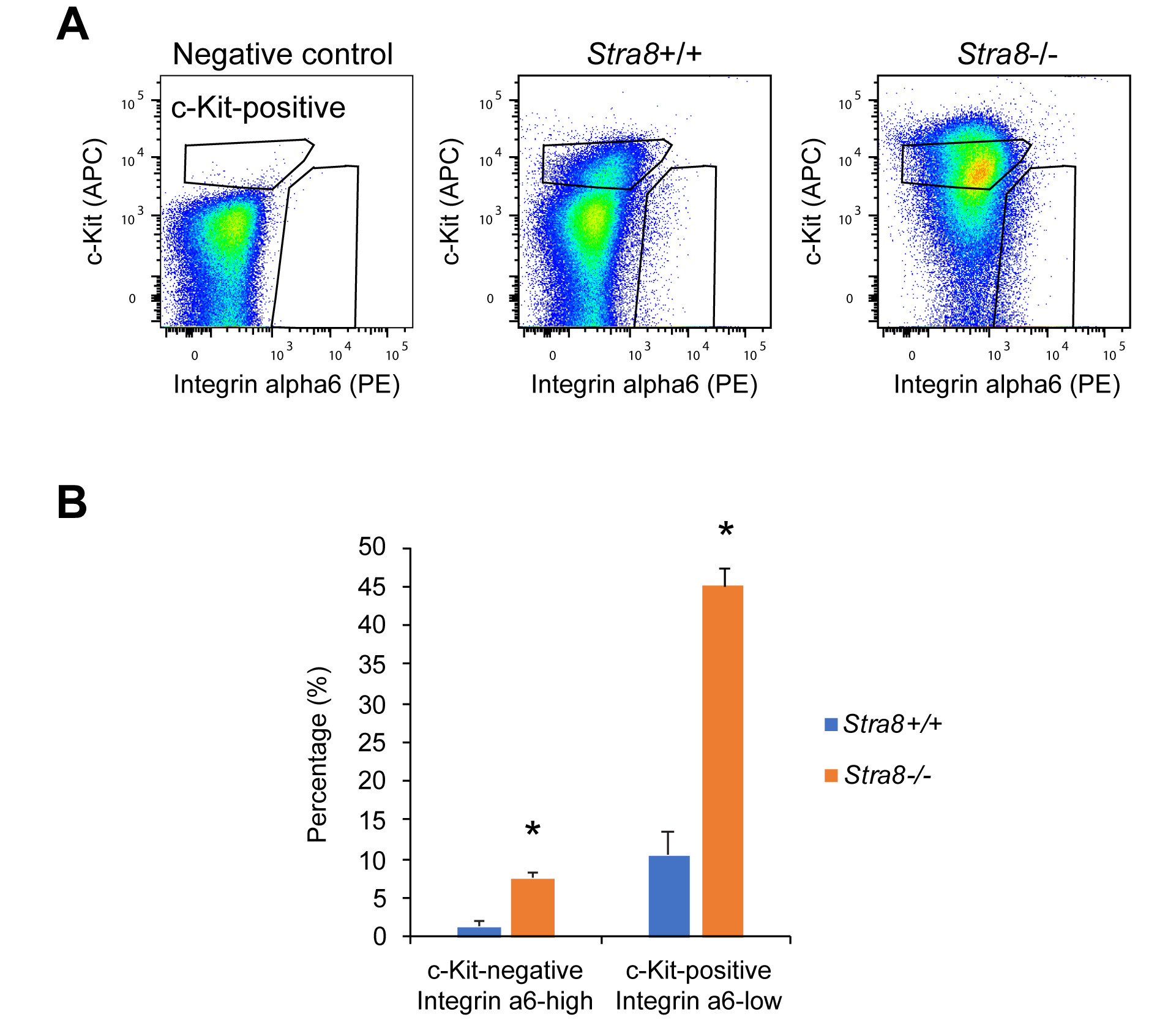

Supplement: S7 Fig — (A) Representative flow profile of dissociated wild-type and Stra8-deficient testicular cells stained with APC-conjugated c-Kit and PE-conjugated integrin α6. (B) quantification of the percentage of c-Kit-negative integrin α6-high and c-Kit-positive integrin α6-low populations in wild-type and Stra8-deficient testes analyzed by FACS in panel (A). Graphs represent mean value ± s.e.m. n = 3 mice per group. *P < 0.05. Both undifferentiated and differentiating spermatogonia exhibited expansion in cellular population in Stra8-deficient testes, probably due to blocked meiotic initiation. (TIF) [file pgen.1008084.s007.tif]

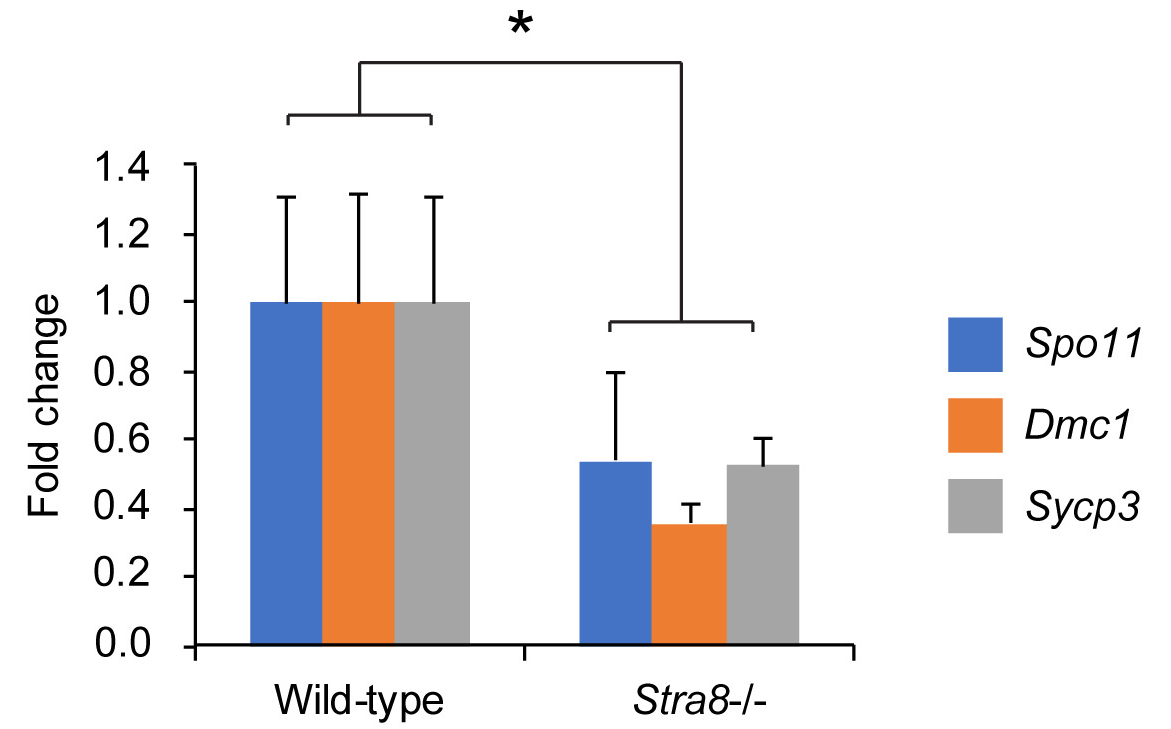

Supplement: S8 Fig — Data represent mean ± SD; n = 5 mice per group; *P < 0.05 (Student’s t test). (TIF) [file pgen.1008084.s008.tif]

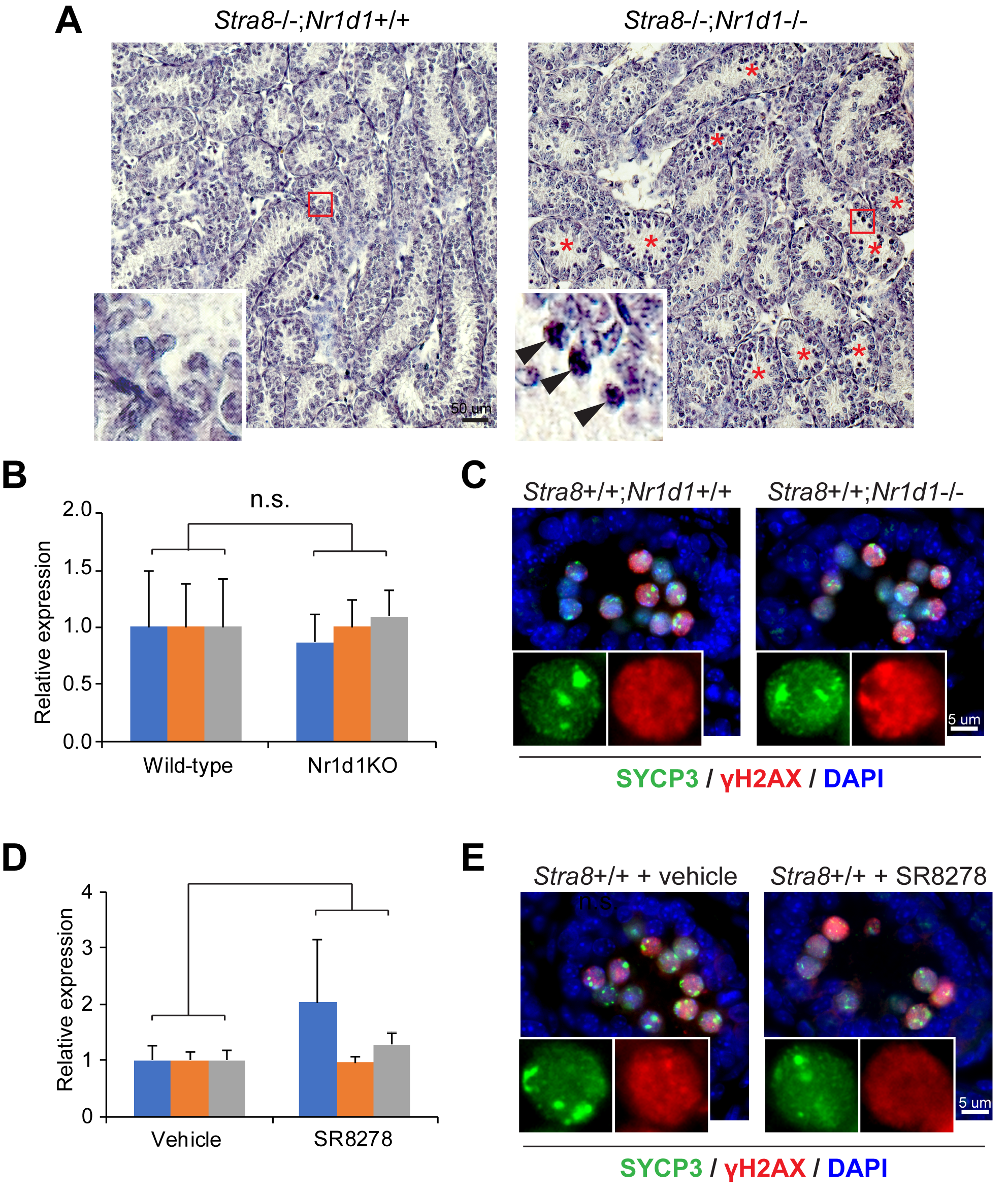

Supplement: S9 Fig — (A) Low power magnification view of Stra8-/-;Nr1d1+/+ and Stra8-/-;Nr1d1-/- testes at 10 d.p.p.. Red asterisks in Stra8-/-;Nr1d1-/- testes indicate tubules containing germ cells with condensed chromosomes. Image in lower left corner shows enlargement of areas in red boxes. Arrow heads show germ cells with condensed chromosomes found in Stra8-/-;Nr1d1-/- testes. (B and D) Quantitative RT-PCR analysis of Spo11, Dmc1, and Sycp3 expression normalized to β-actin in testes with indicated genotypes and treatments. Data are mean ± SD; n = 3–5 mice per group. (C and E) Dual immunofluorescence staining of γ-H2AX and SYCP3 in testes with indicated genotypes and treatments. (TIF) [file pgen.1008084.s009.tif]

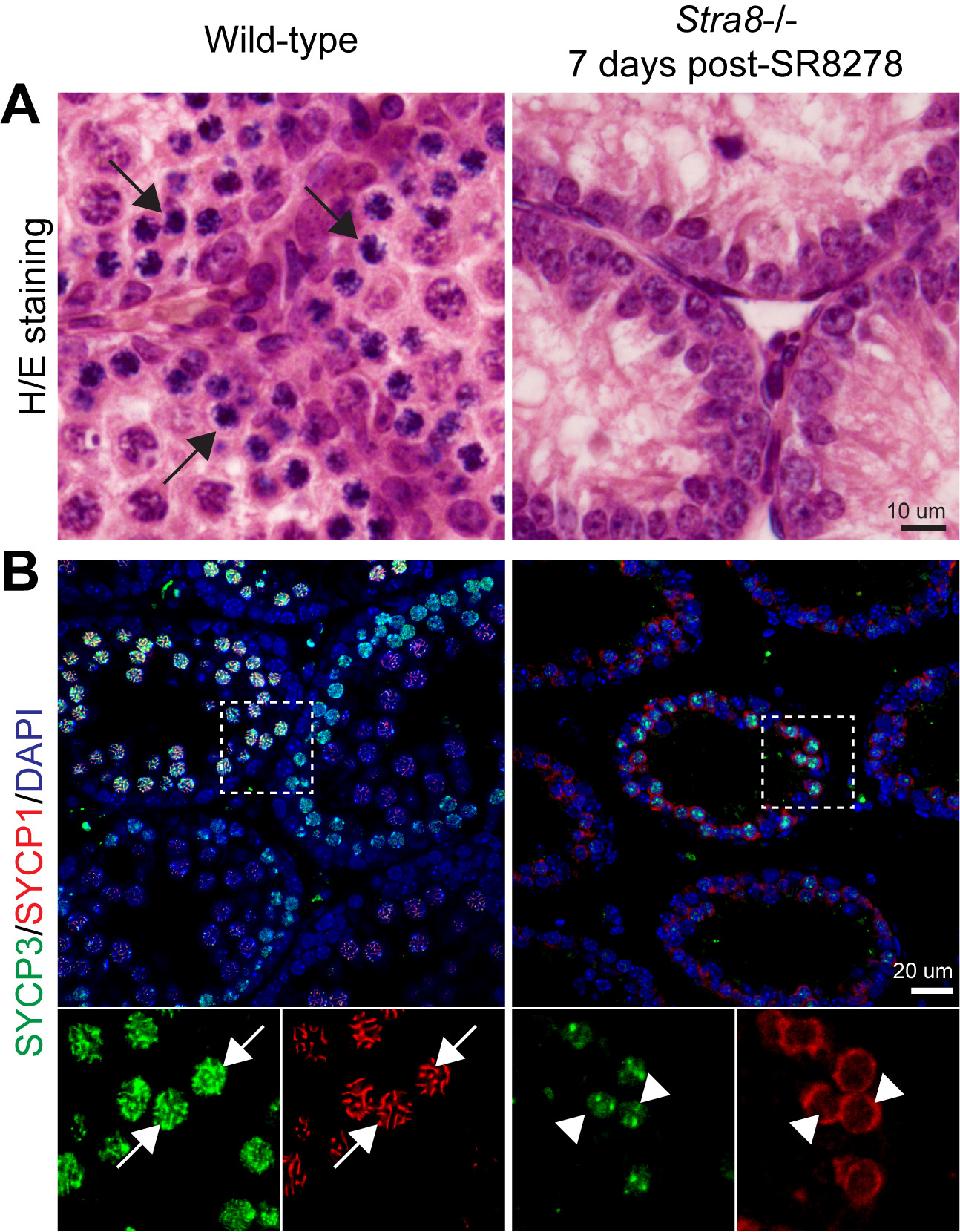

Supplement: S10 Fig — (A) Hematoxylin-eosin staining of testicular sections from age-matched wild-type and Stra8-deficient mice 7 days post-SR8278 treatment. Arrows in wild-type testes indicate meiotic spermatocytes. (B) Immunofluorescence staining for SYCP1/SYCP3 of testicular sections from wild-type and Stra8-deficient mice 7 days post-SR8278 treatment. Enlarged area in wild-type testes show meiotic spermatocytes with synaptonemal complex formation by SYCP3 and SYCP1 (arrows). Enlarged area in Stra8-deficient testes 7 days post-SR8278 show speckle pattern of SYCP3 and cytoplasmic SYCP1 (arrow heads). (TIF) [file pgen.1008084.s010.tif]
